# MOF-Engineered Platelet-Mimicking Nanocarrier-Encapsulated Cascade Enzymes for ROS Scavenging and Anti-Inflammation in Cerebral Ischemia–Reperfusion Injury

**DOI:** 10.3390/pharmaceutics17111478

**Published:** 2025-11-16

**Authors:** Hao Li, Xiaowei Xie, Yu Zhang, Xiaopeng Han, Ting Shi, Jiayin Li, Wanyu Chen, Qin Wei, Hong Pan, Shuxian Xu, Qiuyu Chen, Lifang Yin, Chao Qin

**Affiliations:** 1 Department of Pharmaceutics, China Pharmaceutical University, Nanjing 210009, China; 3122014047@stu.cpu.edu.cn (H.L.);; 2 Key Laboratory of Drug Quality Control and Pharmacovigilance, China Pharmaceutical University, Nanjing 210009, China; 3 State Key Laboratory of Natural Medicine, China Pharmaceutical University, Nanjing 210009, China

**Keywords:** biomimetics, metal–organic frameworks (MOFs), reactive oxygen species scavenging, ischemic stroke, neuroinflammation

## Abstract

**Background/Objectives:** Cerebral ischemia–reperfusion injury (CIRI) remains a major challenge in the treatment of ischemic stroke, characterized by intertwined oxidative stress and neuroinflammation. Existing monotherapies often fail to address this dual pathology effectively. We developed PLSCZ, a biomimetic nanoplatform integrating a catalytic core of imidazolate framework-8 (ZIF-8)-encapsulated superoxide dismutase (SOD) and catalase (CAT) enzymes with a hybrid platelet membrane shell. This design strategically employs metal–organic frameworks (MOFs) to effectively overcome the critical limitations of enzyme instability and provide a cascade catalytic environment, while the biomimetic surface modification enhances targeting capability, thereby enabling dual-pathway intervention against CIRI. **Methods:** PLSCZ was engineered by co-encapsulating SOD and CAT within a ZIF-8 core to form a cascade antioxidant system (SCZ). The core was further coated with a hybrid membrane composed of rapamycin-loaded phospholipids and natural platelet membranes. The nanoparticle was characterized by size, structure, enzyme activity, and targeting capability. In vitro and in vivo efficacy was evaluated using oxygen–glucose deprivation/reoxygenation (OGD/R) models and a transient middle cerebral artery occlusion/reperfusion (tMCAO/r) rat model. **Results:** In vitro, PLSCZ exhibited enhanced enzymatic stability and cascade catalytic efficiency, significantly scavenging reactive oxygen species (ROS) and restoring mitochondrial function. The platelet membrane conferred active targeting to ischemic brain regions and promoted immune evasion. PLSCZ effectively polarized microglia toward the anti-inflammatory M2 phenotype, reduced pro-inflammatory cytokine levels, restored autophagic flux, and preserved blood–brain barrier integrity. In vivo, in tMCAO/r rats, PLSCZ markedly targeted the ischemic hemisphere, reduced infarct volume, improved neurological function, and attenuated neuroinflammation. **Conclusions:** By synergistic ROS scavenging and anti-inflammatory action, the PLSCZ nanozyme overcomes the limitations of conventional monotherapies for CIRI. This biomimetic, multi-functional platform effectively reduces oxidative stress, modulates the phenotype of microglia, decreases infarct volume, and promotes neurological recovery, offering a promising multi-mechanistic nanotherapeutic for CIRI and a rational design model for MOF-based platforms.

## 1. Introduction

Ischemic stroke, a leading cause of global mortality and disability, is increasingly prevalent with population aging [1,2]. While rapid reperfusion is the standard treatment [3], it often induces cerebral ischemia–reperfusion injury (CIRI), characterized by oxidative stress and neuroinflammation [4,5,6]. These processes form a vicious cycle, exacerbating neuronal damage and blood–brain barrier (BBB) disruption [7,8]. Thus, dual modulation of oxidative stress and neuroinflammation represents a critical therapeutic frontier.

The cascade of reactive oxygen species (ROS), such as O_2_^−^, OH, and H_2_O_2_, drives secondary injury following stroke [9]. Through oxidative damage to key cellular components, ROS promotes neuronal death, inflammatory activation, and BBB disruption, fueling a cycle of progressive brain damage [10,11].To counteract oxidative damage in stroke, a variety of strategies have been developed to scavenge ROS [12]. However, their practical efficacy is often limited by inefficient ROS clearance compared with natural antioxidant enzymes [13], short half-life [14], long-term toxicity, or rapid metabolic clearance of the antioxidants [15,16]. In contrast, natural antioxidant enzymes, specifically superoxide dismutase (SOD) and catalase (CAT), constitute a primary biological defense by operating in a concerted catalytic cascade: SOD converts superoxide into H_2_O_2_, which CAT then decomposes into harmless water and oxygen [17,18]. However, their efficacy as free enzymes is limited by instability, short half-life, and inefficient cascade kinetics due to lack of co-localization [19].

Metal–organic frameworks (MOFs), which are porous frameworks formed by the coordination bonding of metals and organic compounds, provide excellent solutions to these problems, particularly zeolitic imidazolate framework-8 (ZIF-8), which is directly synthesized under gentle aqueous conditions [20,21]. MOFs have found success in enzyme immobilization for diverse applications such as antitumor therapy [22], anti-inflammation [23], and wound healing [24]. However, their potential in ischemic stroke has been rarely reported. This confined environment of MOFs not only protects the enzymes but also enhances catalytic efficiency by facilitating substrate channeling between SOD and CAT [25,26]. The synergistic co-delivery of SOD/CAT represents a promising strategy for treating CIRI, yet instability and poor targeting hinder its application. Furthermore, covalent modification has been the predominant approach for the targeted modification of MOFs in previous research [27]. Nevertheless, this strategy not only poses biocompatibility problems for the synthetic products but may also impair the crystal structure of MOFs, leading to failed targeting or unsuccessful delivery.

To address this, platelet membranes (PMs) have been used to modify the surface of MOFs to enhance their low systemic cytotoxicity, prolonged blood circulation time, and excellent biodegradability by mimicking natural cell interactions [28], thereby overcoming the main challenges of the drug delivery system [29]. These platelet membranes leverage platelet-specific surface markers (e.g., GPIbα and CD47) for targeted delivery to injured ischemic cerebral vasculature. The platelet membrane modification further enhances immune evasion and promotes penetration into damaged brain regions [30,31,32]. In addition, we further modified the platelet membrane to prepare an innovative hybrid membrane. By fusing platelet membranes with phospholipids, we successfully encapsulated rapamycin (RAPA), which has shown remarkable potential in reducing neuroinflammation and regulating the polarization of microglia [33]. This strategy overcomes clinical limitations of rapamycin, such as poor bioavailability and systemic toxicity [34,35], and achieves targeted and synergistic anti-inflammatory therapy to address the intertwined pathways of oxidative stress and neuroinflammation in cerebral ischemia–reperfusion injury.

**Figure 1:**
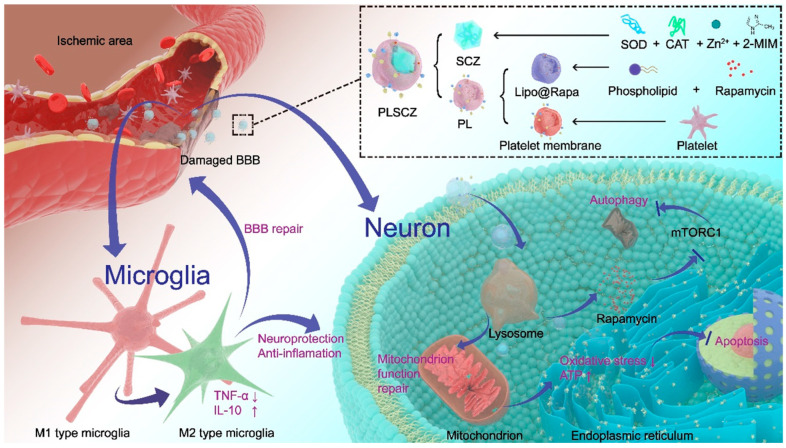
Schematic illustration of PLSCZ (a biomimetic nanoplatform integrating a catalytic core of imidazolate framework-8 (ZIF-8)-encapsulated superoxide dismutase (SOD) and catalase (CAT) enzymes with a hybrid platelet membrane shell.) for targeted antioxidant and anti-inflammatory therapy in cerebral ischemia–reperfusion injury.

This study introduces PLSCZ, a biomimetic nanoplatform engineered for the comprehensive treatment of CIRI by synergistically combining a cascade-enzymatic ZIF-8 core with a functional platelet membrane shell (Figure 1). Its design incorporates several key innovations: By co-encapsulating SOD and CAT within a ZIF-8 framework, this system simultaneously stabilizes the enzymes—boosting their cascade efficiency—and addresses their delivery challenges, thus fully unlocking the therapeutic potential of natural antioxidant defense. Furthermore, PLSCZ uniquely integrates complementary antioxidant and anti-inflammatory capabilities within a single agent, enabling simultaneous intervention against the two primary, interconnected pathological pathways of CIRI. This dual-therapy approach offers a more comprehensive and potent therapeutic effect than conventional single-target strategies. Finally, the platelet membrane coating strategically addresses the drawbacks of MOFs, enabling targeted delivery and immune evasion. This synergistic design not only establishes a comprehensive and biomimetic therapy for CIRI but also highlights the considerable potential of MOF-based nanocarriers for ischemic stroke, thereby enriching the scope of MOF research in biomimetic nanocarrier design and CIRI therapeutics.

## 2. Materials and Methods

### 2.1. Materials

Rapamycin (F21575) was purchased from MERYER (Shanghai, China). Prostaglandin E1 (P129960), phenylmethyl sulfonyl fluoride (P105539), pyrogallol (P104234), triphenyl tetrazolium chloride (T109275), cholesterol (C104028), zinc nitrate hexahydrate (Z111703) chlorpromazine (C184839), methyl-β-cyclodextrin (M1421232), nystatin (N141226), colchicine (C425287), dimethyl sulfoxide (D670381) and riboflavin-5‘-phosphate (F107158) were purchased from Aladdin (Shanghai, China). Superoxide dismutase (S9697) and catalase (C40) were from Sigma-Aldrich (St. Louis, MO, USA). 2-Methylimidazole (M813135) was purchased from Macklin (Shanghai, China). Egg phospholipids (PC-98T) were purchased from AVT Pharmaceutical Technology (Shanghai, China). Thrombin (S10117) and Fibrinogen (S12024) were purchased from Shanghai Yuanye Bio-Technology (Shanghai, China).

DAPI (C1006), One-Step TUNEL Apoptosis Assay Kit (C1086), H&E staining kit (C0105S), RIPA lysis solution (P0013B), BCA protein quantification kit (P0010), DCFH-DA test kit (S1105S), Mitochondrial Membrane Potential assay kit with TMRE (C2001S), Mito-Tracker Red CMXRos (C1035), DIO (C1038), DIR (Y239280), and Lyso-Tracker Red (C1046) were purchased from Beyotime (Shanghai, China). ER-Tracker Blue-White DPX (40761ES) was purchased from Yeasen Biotechnology (Shanghai, China). Annexin V-FITC/PI apoptosis detection kit (KGA1102), Dulbecco’s modified Eagle’s medium/F12 (KGL1202), streptomycin and penicillin (KGL2304), trypsin (KGL2101), fetal bovine serum (KGL3006), and enhanced chemiluminescence (KGC4603) were purchased from KeyGEN BioTECH (Nanjing, China). TNF-α Elisa kit (E-EL-R2856) and IL-10 Elisa kit (E-EL-R0016) were purchased from Elabscience (Wuhan, China). PVDF membranes (IPVH00010) were purchased from Millipore (Billerica, MA, USA).

HRP-conjugated Goat Anti-Rabbit IgG(H+L) (SA00001-2), CD41 antibody (24552-1-AP), STAT3 antibody (3G2D12), p-STAT3 antibody (2G11G11), NF-κB p65 antibody (4C7), p62 antibody (241992C4), occludin antibody (1D3C4), CoraLite488-conjugated Goat Anti-Mouse IgG(H+L) (SA00013-1), CoraLite594–conjugated Goat Anti-Rabbit IgG(H+L) (SA00013-4), CoraLite488-conjugated Goat Anti-Rabbit IgG(H+L) (SA00013-2), β-actin antibody (2D4H5), APC-CD86 antibody (230476A1), FITC-CD206 antibody (240344B9), Iba-1 antibody (4D5), GFAP antibody (4B2E10) and NeuN antibody (3A4C1) were purchased from Proteintech (Wuhan, China). NcmColor marker (14kDA-250kDA) (P9006,P9001) was purchased from NCM Biotech (Suzhou, China).

### 2.2. Cell Lines and OGD/R Model

Human neuroblastoma cells (SH-SY5Y, CL-0208) were grown in Dulbecco’s modified Eagle’s medium/F12 (DMEM/F12) containing 10% FBS, 100 mg/mL streptomycin, and 100 U/mL penicillin. Mouse microglia cells (BV2, CL-0493) were grown in DMEM medium containing 10% FBS, 100 mg/mL streptomycin, and 100 U/mL penicillin. Mouse monocyte macrophage leukemia cells (RAW 264.7, CL-0190) were grown in DMEM medium containing 10% FBS, 100 mg/mL streptomycin, and 100 U/mL penicillin. Mouse brain-derived endothelial cells (bEnd.3, CL-0598) were grown in DMEM medium containing 10% FBS, 100 mg/mL streptomycin, and 100 U/mL penicillin. All cells were purchased from Pricella Biotechnology (Wuhan, China).

Cells were incubated at 37 °C under a saturating humidity atmosphere containing 5% CO_2_. For the oxygen–glucose deprivation/reoxygenation (OGD/R) model, cell culture medium was replaced with low-glucose DMEM, and the cells were then placed in an anaerobic atmosphere containing 94% N_2_, 1% O_2_, and 5% CO_2_ for a certain amount of time, followed by normal culture conditions [36].

### 2.3. Animals and MCAO Model

Male Sprague–Dawley (SD) rats (~200 g) aged 6–8 weeks were purchased from the Kasiwen Biotech (Nanjing, China; License Number: SCXK(Su)2021-0013). In order to ensure a sufficient number of samples for analysis, animals were allocated as follows: platelet donor rats (*n* = 6), the transient middle cerebral artery occlusion/reperfusion (tMCAO/R) model for neuroprotection evaluation and related mechanistic studies (*n* = 105), and the tMCAO/R model for targeting and biodistribution studies (*n* = 9). The specific grouping and the arrangement of the number of rats are included in the detailed experimental description below.

The use of animals was necessary as in vitro models cannot fully replicate the complex pathophysiology of cerebral ischemia–reperfusion injury, including neuroinflammation, blood–brain barrier dynamics, and systemic responses, which are critical for evaluating the therapeutic efficacy and targeting capability of the developed nanozyme. Animals were housed under individually ventilated cage (IVC) conditions in the animal experiment center of China Pharmaceutical University (License Number: SCXK(Su)2023-0019) with ad libitum access to food and water. The positions of the animals and cages were randomly arranged. All surgical procedures and post-operative care were performed with strict adherence to aseptic techniques. All animal experiments were conducted according to the protocol approved by the Laboratory Animal Ethics Committee of China Pharmaceutical University (protocol code 2024-06-081; approval date: 22 June 2024) and in accordance with the ARRIVE guidelines.

The transient middle cerebral artery occlusion/reperfusion (tMCAO) model was established according to a previous protocol [37]. Rats were anesthetized with isoflurane (induction at 4–5%, maintenance at 2–3% in oxygen). The external carotid artery (ECA) was exposed, and the monofilament was inserted into the internal carotid artery (ICA) through the ECA until it reached the middle cerebral artery (MCA), causing a blockage of blood flow. The MCAO monofilament was gently withdrawn 2 h later for reperfusion. Rats having the same procedure without monofilament blocking were used as the sham group. According to Long’s scoring [37], rats with significant neurological functional impairments (scores ≥ 2) were included in the sample. For terminal procedures, rats were euthanized by exposure to a gradually filled chamber with compressed carbon dioxide (CO_2_) at a displacement rate of 30% to 70% of the chamber volume per minute, followed by decapitation to ensure death.

### 2.4. Platelet Isolation and Membrane Extraction

Platelet isolation and membrane extraction were performed according to a previous protocol [30]. Whole blood was collected via the orbital venous plexus from male SD rats. All blood collection procedures were conducted under brief isoflurane anesthesia (induction at 4–5% and maintenance at 2–3% in oxygen) to ensure profound anesthesia and minimize discomfort. Approximately 0.5 mL of blood was collected per session from each animal. This volume and the sampling schedule were designed to comply with animal welfare guidelines, allowing sufficient time for recovery and physiological restoration between collections. The specific interval between blood collections was 2 weeks to ensure animal well-being and hematological recovery. Endpoint was established where each animal was euthanized by CO_2_ asphyxiation after a maximum of three blood collections.

Briefly, whole blood was collected from the orbital veins of male Sprague–Dawley (SD) rats into tubes containing 10% anticoagulant citrate dextrose (ACD) buffer. Sequential centrifugation steps were performed: initial centrifugation at 100× *g* for 20 min (4 °C) to pellet erythrocytes, followed by centrifugation of the supernatant at 800× *g* for 20 min (4 °C) to isolate platelets. The platelets were resuspended in PBS (0.1 M, pH = 7.4) containing prostaglandin E1 (1 × 10^−6^ M) and phenylmethyl sulfonyl fluoride (1 × 10^−5^ M) to inhibit activation and proteolysis. Platelet membranes were extracted by three freeze–thaw cycles (−80 °C to 37 °C), followed by centrifugation at 12,000× *g* for 20 min (4 °C). Membranes were resuspended in 5% glucose and stored at −80 °C.

### 2.5. Synthesis and Characterization of PLSCZ

Briefly, rapamycin (RAPA) (2 mg), cholesterol (1 mg), and egg phosphatidylcholine (EPC, 30 mg) were dissolved in 4 mL chloroform, evaporated under vacuum (37 °C, 60 min), and hydrated with 5% glucose solution. The suspension was sonicated (100 W, 10 min, 4 °C) to form liposomes. For membrane fusion, platelet vesicles (sonicated for 10 min) were mixed with rapamycin-loaded lipid membrane and extruded through a 0.22-µm polycarbonate membrane (30 cycles), followed by sonication (100 W, 30 min, 4 °C).

A total of 2 mL of aqueous solutions containing 2-methylimidazole (256 mg), SOD (2 mg), and CAT (2mg) were mixed and stirred (4 °C, 3 min). Zn(NO_3_)_2_ solution (200 μL, Zn^2+^:2-methylimidazole (*n*/*n*) = 1:70) was added dropwise under vigorous stirring (30 min, 4 °C). SCZ nanoparticles were collected by centrifugation (12,000× *g*, 10 min) and washed with deionized water. SZ (SOD@ZIF-8) and CZ (CAT@ZIF-8) were synthesized analogously by omitting CAT or SOD, respectively. ZIF-8 was prepared using the same method without adding SOD and CAT. SCZ was mixed with rapamycin-loaded lipid membrane or platelet–lipid fusion membrane (*w*/*w* = 1:1), followed by extrusion (0.22 µm membrane, 30 cycles) and sonication (100 W, 1 h, 4 °C) to obtain LSCZ or PLSCZ. PLZIF-8 was prepared using the same method as ZIF-8 and platelet–lipid fusion membrane.

Nanoparticle morphology was analyzed via transmission electron microscopy (HT7700, Hitachi, Tokyo, Japan). The size distribution was measured by DLS (NanoBrook Omni, New York, NY, USA). Zeta potential was measured by a zeta potential detector (Zetasizer Nano ZS90, Malvern, UK). Elemental composition was confirmed by energy-dispersive X-ray spectroscopy (EDS). DIO (1 μg/mL) and DIR (1 μg/mL) were used to label the platelet membrane and rapamycin-loaded lipid membrane, respectively. PLSCZ was synthesized, then diluted in glycerol/water (7:3, *v*/*v*). and photographed by CLSM (LSM700, Zeiss, Jena, Germany).

### 2.6. Hemolysis Assay

A fresh red blood cell (RBC) suspension was prepared using RBCs isolated during the platelet extraction procedure described in Section 2.4. The RBC suspension (50 μL, 16% *v*/*v* in saline) was incubated with 1 mL of ZIF-8 or PLZIF-8 at varying concentrations at room temperature for 1 h. After incubation, the mixtures were centrifuged at 1000× *g* for 5 min, and 200 μL of each supernatant was transferred to a 96-well plate. Absorbance was measured at 540 nm using a microplate reader (Multiskan FC, Thermo, USA). Deionized water and phosphate-buffered saline (PBS) served as the positive control (100% hemolysis) and negative control, respectively. The hemolysis rate was calculated relative to the positive control.

### 2.7. Cytotoxicity Assay

SH-SY5Y cells (1 × 10^4^ cells/well) were seeded in 96-well plates and cultured in DMEM supplemented with 10% FBS at 37 °C under 5% CO_2_ until adherence. The medium was then replaced with fresh medium containing varying concentrations of ZIF-8 or PLZIF-8 suspensions. After 24 h of incubation, cell viability was assessed using the MTT assay [38]. Briefly, MTT solution (0.5 mg/mL) was added to each well and incubated for 4 h. The resulting formazan crystals were dissolved in dimethyl sulfoxide (DMSO), and the absorbance was measured at 490 nm using a microplate reader (Multiskan FC, Thermo, Waltham, MA, USA).

### 2.8. Enzyme Activity Assays

SOD activity has been investigated based on SOD’s ability to inhibit the autoxidation rate of pyrogallol, which was used to measure the SOD activity [39]. In brief, 10 µL of the test sample or blank and 10 µL of the 0.1 M hydrochloric acid solution of pyrogallol were added to 3 mL of Tris-HCL (0.1 M, pH = 8.2, 2 mM EDTA), quickly mixed, and the absorbance of the test sample and the blank was measured at a wavelength of 325 nm for 180 s (UV2600A spectrophotometer, Shimazu, Japan). The ratio of the absorbance change rate between the blank and the sample to be tested was used as the relative activity of SOD, which reflects the O_2_^−·^ scavenging capacity.

CAT activity was measured by monitoring H_2_O_2_ at 240 nm (UV2600A spectrophotometer, Shimadzu, Kyoto, Japan). The assay principle is based on the reaction of catalase with H_2_O_2_. The solution’s adsorption at 240 nm can be used to calculate the concentration of H_2_O_2_ and, therefore, to indirectly indicate the CAT activities. In brief, 200 µL of the sample or blank and 200 µL of 0.3% H_2_O_2_ were added to 2.8 mL of PBS (0.1 M, pH = 7.4), quickly mixed, and the absorbances of the sample and the blank were determined at a wavelength of 240 nm for 180 s. The ratio of the absorbance change rate between the sample and the blank was used as the relative activity of CAT.

In order to investigate the protective effect of the preparation on the enzyme activity, free CAT and CZ were incubated in rat serum or PBS (0.1 M, pH = 7.4) containing trypsin (0.05, 0.1, 0.5 μg/mL) at 37 °C for 24 h. Then, the CAT activity was detected by the method above.

### 2.9. Evaluation of Antioxidant Stress and Neuroprotection In Vitro

SH-SY5Y cells (1 × 10^4^ cells/well) were seeded in 96-well plates and subjected to OGD/R followed by 24 h reoxygenation. Different formulations were administered at the beginning of reoxygenation. Cell viability was assessed via MTT assay as described in Section 2.7. Intracellular ROS levels were measured using DCFH-DA fluorescence. The resulting 2’,7’-dichlorodihydrofluorescein (DCFH) is proposed to react with intracellular hydrogen peroxide or other oxidizing ROS to give the fluorescent 2’,7’-dichlorofluorescein (DCF) [40]. Briefly, SH-SY5Y cells (1 × 10^5^ cells/well) were seeded in 6-well plates or confocal dishes and subjected to OGD/R followed by 24 h reoxygenation. Subsequently, 100 μL of DCFH-DA dye working solution was added to cells, gently shaken to completely cover the cells, and incubated for 5-30 min. The dye working solution was removed by aspiration, and the cells were washed with DMEM medium 2-3 times for 5 min each, before being visualized using fluorescence microscopy (LSM700, Zeiss, Germany) or flow cytometry (CytoFlex, Beckman, Boulevard Brea, CA, USA).

### 2.10. Microglial Polarization Analysis

BV2 cells (5 × 10^5^ cells/dish) were seeded in 6-well plates or confocal dishes and subjected to OGD/R followed by 24 h reoxygenation with different treatments. Cells were immunostained by APC-CD86 antibody (1:200) and FITC-CD206 antibody (1:1000), before being analyzed by confocal microscopy (LSM700, Zeiss, Germany) and flow cytometry (CytoFlex, Beckman, USA). Cytokine levels (TNF-α, IL-10) were quantified via ELISA kits according to the manufacturer’s instructions.

### 2.11. Cellular Uptake and Intracellular Trafficking

SH-SY5Y (1 × 10^5^ cells/dish) and BV2 cells (1 × 10^5^ cells/dish) were seeded in 6-well plates and confocal dishes. FITC-labeled CAT or C6 was used to further prepare the formulations using the methods described at 2.5. Subsequently, different formulations were incubated with SH-SY5Y or BV2 cells (for 4 and 12 h). Cell uptake was investigated by flow cytometry (CytoFlex, Beckman, Boulevard Brea, CA, USA). Cell uptake and organelle colocalization were further assessed using Lyso-Tracker Red (60 nM), Mito-Tracker Red (100 nM), and ER-Tracker Blue (100 nM) by confocal microscopy (LSM700, Zeiss, Germany).

To investigate the cellular uptake mechanism, SH-SY5Y (1 × 10^5^ cells/dish) and BV2 cells (1 × 10^5^ cells/dish) were seeded in 6-well plates. Chlorpromazine (CPZ, 10 μg/mL, an inhibitor of clathrin-mediated endocytosis), methyl-β-cyclodextrin (M-CD, 2.5 mM, an inhibitor of caveolin-mediated endocytosis), nystatin (10 μM, an inhibitor of the lipid raft pathway), colchicine (10 μM, an inhibitor of macropinocytosis), and 4 °C treatment (inhibition of energy-related endocytic pathway) were applied to the pretreatment of SH-SY5Y and BV2 cells. After 4 h of incubation with SCZ, LSCZ, and PLSCZ containing CAT-FITC, the uptake was analyzed by flow cytometry (CytoFlex, Beckman, Boulevard Brea, CA, USA).

To investigate immune escape, RAW 264.7 (1 × 10^5^ cells/dish) were seeded in 6-well plates. After 4 h of co-incubation with different formulations containing CAT-FITC, the uptake by RAW 264.7 was analyzed by flow cytometry (CytoFlex, Beckman, Boulevard Brea, CA, USA).

### 2.12. In Vitro and In Vivo Targeting and Permeability

The tMCAO model was induced in SD rats using the methods described in Section 2.5. The model rats were further verified by a laser speckle imaging system (MoorFLPI-2, Moor instrument, Axminster, UK). To obtain fluorescence-labeled nanoparticles, riboflavin-5’-phosphate was incorporated during the preparation of SCZ. The rats were subjected to MCAO for 2 h followed by reperfusion. They were randomly divided into 3 groups with intravenous injection (100 μL/g) of riboflavin-5’-phosphate-labeled SCZ, LSCZ, and PLSCZ (*n* = 3/group). The animals were sacrificed 4 h later, and the brain and major organs were harvested for fluorescence imaging by the In Vivo Imaging System (IVIS) (ABL-X6, Tannon, Shanghai, China). To investigate the clot-targeting property of PLSCZ, an artificial clot gel was constructed in vitro according to previous studies [36]. Briefly, 50 μL fibrinogen (10 mg/mL), 15 μL thrombin (6 U/mL), and 5 μL CaCl_2_ (200 mM) were mixed with 40 μL HEPES buffer (20 mM, pH 7.4) in 96-well plates and incubated at 37 °C for 30 min to form clots. A total of 20 μL of riboflavin-5’-phosphate-labeled SCZ, LSCZ, and PLSCZ was then added to the clots and incubated at 37 °C for another 60 min. The clots were washed intensively, before which the fluorescence was monitored and quantified by the In Vivo Imaging System (IVIS) (ABL-X6, Tannon, Shanghai, China). To investigate the BBB permeability, b.End.3 cells were seeded at 5 × 10^4^ cells/well in Transwell inserts (0.4 μm PET membrane; Biofil, Guangzhou, China) with 200 μL complete DMEM medium, while the lower chamber contained 600 μL complete DMEM medium. After 5 days of culture with daily medium changes, tight monolayer formation was confirmed by stabilized TEER values measured by an epithelial volt–ohmmeter (RE1600, Jingonghongtai, Beijing, China) [41]. The monolayers were then divided into two groups: control and OGD/R. The OGD/R group was subjected to 2 h of OGD induction, followed by return to normal culture conditions. Riboflavin-5’-phosphate-labeled PLSCZ was added to the upper chamber and incubated for 2 h under normal conditions. Permeability was quantified by measuring the absorbance of the lower chamber medium at 460 nm using a microplate reader (Multiskan FC, Thermo, Waltham, MA, USA). Results were expressed as the permeability ratio of the OGD/R group to the normal group.

### 2.13. Neuroprotection Evaluation

The tMCAO model was induced in SD rats. The reperfusion was performed 2 h later with intravenous administration. Rats were randomized into 8 groups (n = 6/group) and received intravenous injections (100 μL/g) of 5% glucose (sham and tMCAO/r), free SOD/CAT, SCZ, rapamycin, SCZ and RAPA, LSCZ, or PLSCZ. The treated rats were allowed another 24 h of reperfusion before sacrifice. Brains were immediately removed and used for TTC staining, which was performed as reported in previous studies [42]. The treated rats were anesthetized and sacrificed after 24 h of reperfusion. Brains were immediately removed and further fixed with 4% paraformaldehyde for 24 h, followed by dehydration with 15% and 30% sucrose stepwise at 4 °C overnight. Then, 20 μm brain sections were prepared. Cell apoptosis in ischemic penumbra was stained with One-Step TUNEL Apoptosis Assay Kit according to the manufacturer’s instructions. Brain sections were stained using the H&E staining kit, following the instructions.

Treated rats underwent behavioral testing (*n* = 6/group) at days 1, 2, 4, 6, 8, 10, 12, and 14 after the MCAO model. Neurological deficits were evaluated using the modified Neurological Severity Score (mNSS) as previously described [43]. This scoring system grades neurological function on a scale of 0 (normal) to 18 (maximal deficit); thus, higher scores indicate more severe injury. For the adhesive-removal somatosensory test, somatosensory function was assessed postoperatively, as previously described [44]. Prior to surgery, rats underwent training for 3 days. Rats that consistently removed the dots within 10 s were then subjected to MCAO. All rats were acclimated to the testing environment prior to assessment. In this test, two small, adhesive-backed paper dots (each 113.1 mm^2^) were applied bilaterally to the distal-radial region of each forelimb as tactile stimuli. The rat was then returned to its cage, and the time taken to remove each stimulus from the forelimbs was recorded. Each rat performed three trials, with individual trials separated by at least 5 min on days 1, 3, 5, 7, and 14.

### 2.14. Immunofluorescence Staining

Briefly, rats were sacrificed 24 h after administration and reperfusion following the MCAO model as described before. Brain tissues were harvested and immersed in paraformaldehyde (4%), and coronal paraffin blocks were subsequently prepared. Specific coronal sections were subsequently sectioned and deparaffinized. Sections were placed in boiled citrate buffer (pH 6.0) within a microwave oven (650 to 720 W). After blocking in normal serum, sections were treated with the following primary antibodies: APC-CD86 antibody (1:200), FITC-CD206 antibody (1:1000), Iba-1 antibody (1:1000), GFAP antibody (1:200), and NeuN antibody (1:1000). Secondary antibodies, including Goat anti-rabbit IgG Alexa Flour 488, Goat anti-rabbit IgG Alexa Flour 594 and Goat anti-mouse IgG Alexa Flour 488, were applied with 1:1000 dilution, followed by DAPI staining before fluorescence imaging under a fluorescence microscope (Leica, Solms, Germany).

### 2.15. Western Blotting

Rats were sacrificed 24 h after administration and reperfusion following the MCAO model as described before. Brain tissues were collected. The ischemic penumbra cortex was isolated and homogenized in RIPA lysis buffer. Tissue lysates were centrifuged at 13,500× *g* for 5 min at 4 °C, and the supernatant was collected for protein quantification. Total protein (40 µg per sample) was separated by 7.5% SDS-PAGE and electrotransferred onto PVDF membranes. Membranes were blocked with 5% bovine serum albumin (BSA) and then probed overnight at 4 °C with primary antibodies against phospho-STAT3 (p-STAT3, 1:1000), NF-κB p65 (1:1000), p62 (1:1000), occludin (1:1000), and β-actin (1:1000). Following primary antibody incubation, membranes were incubated with horseradish peroxidase (HRP)-conjugated secondary antibodies at room temperature for 2 h. Protein bands were visualized using enhanced chemiluminescence (ECL) reagents and detected using the Image Lab software (Bio-Rad, Hercules, CA, USA). Band intensities were quantified by densitometry using Image Lab and normalized to β-actin expression levels for further analysis. Representative Western blot images shown are from one animal per group, consistent across three independent experiments.

### 2.16. Statistical Analysis

The sequence of sample processing and measurement was random, and the testers were unaware of the sample groups when conducting the testing experiments. Results were analyzed by the GraphPad Prism software (10.1.2(324), GraphPad Software, San Diego, CA, USA) and presented as mean ± SD. The statistical significance was evaluated by Student’s t-test and one-way ANOVA. A P value < 0.05 was considered statistically significant. All the images of fluorescence immunostaining are from one representative of three separate experiments. The imaging area was defined as the ischemic penumbra, which was the border region of the ischemic brain area. At least 3 imaging fields were randomly chosen, and fluorescence intensity or positive cells were quantified with ImageJ (1.53t, National Institudes of Health, Bethesda, MD, USA) for further analysis. All the data were obtained during the study period. This study was conducted from February 2022 to July 2025.

## 3. Results

### 3.1. Preparation and Characterization of SZ, CZ, SCZ, PLSCZ

**Figure 2:**
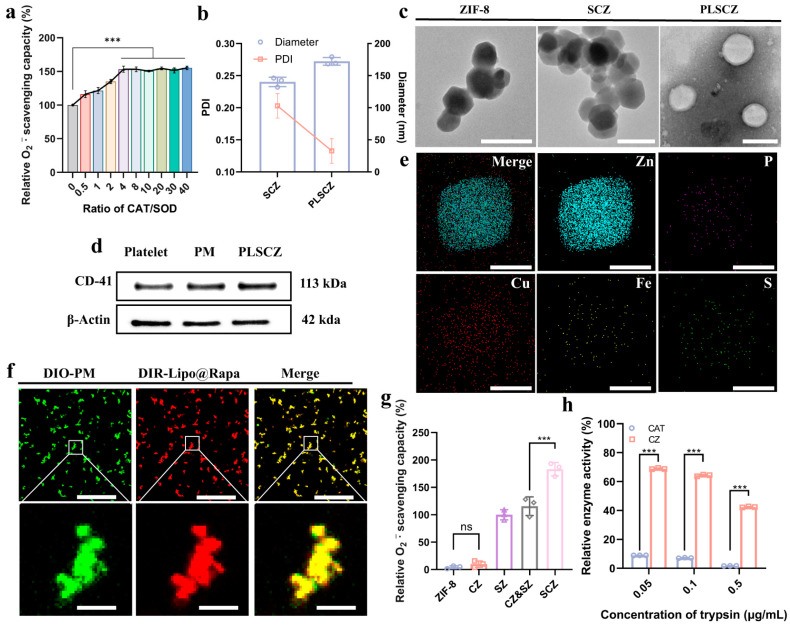
Characterization of PLSCZ nanoparticles. (**a**) Relative O_2_^−·^ scavenging capacity of a physical mixture of SOD/CAT with different mass ratios. (**b**) Particle sizes of different nanoparticle formulations measured by DLS. (**c**) Representative image of the morphology of different nanoparticle formulations observed with TEM ( the image of PLSCZ was used, as well as the Negative-Staining method by phosphotungstic acid) (scale bar: 200 nm). (**d**) Representative of Western blot bands of CD41 of the platelet, platelet membrane, and PLSCZ. (**e**) EDS mapping of elemental distributions (scale bar: 100 nm). (**f**) CLSM images of PLSCZ; platelet membrane was labeled with DIO (green), and Rapa@lipo was labeled with DIR (red); scale bar: 10 µm (up), 1 µm (down). (**g**) Relative O_2_^−·^ scavenging capacity of SCZ, SZ, CZ, ZIF-8, or single-loaded nanoparticles (SZ and CZ). (**h**) Protease stability assays. Data presented as mean ± SD (*n* = 3); ns *p* > 0.05, *** *p* < 0.001.

SOD and CAT function synergistically to neutralize intracellular ROS [45]. SOD converts superoxide into H_2_O_2_, which CAT subsequently decomposes into water and oxygen [46,47]. To optimize this cascade, we evaluated various mass ratios of SOD and CAT and identified a 4:1 (CAT:SOD) ratio as optimal, enhancing SOD activity by 153.24 ± 3.71% compared to free enzymes (Figure 2a). This ratio significantly enhances the clearance efficiency of O_2_^−·^. SOD has little effect on the decomposition of H_2_O_2_ catalyzed by CAT (Figure S1), indicating the direction of catalysis and the cascade mechanism.

To further enhance stability and cascade properties of enzymes, SOD and CAT were co-encapsulated within a ZIF-8 framework via a one-pot coprecipitation method, forming the SCZ nanozyme. Subsequently, a hybrid membrane—composed of rapamycin-loaded lipids fused with platelet membrane—was coated onto SCZ, yielding the biomimetic nanoparticle PLSCZ. Dynamic light scattering (DLS) confirmed successful fusion, showing increased hydrodynamic diameter (172.3 ± 4.9 nm) and reduced polydispersity (PDI = 0.13) compared to SCZ (Figure 2b). The zeta potential shifts from +26.3 mV (ZIF-8) to −11.5 mV (PLSCZ), further verified by surface coating (Figure S2). TEM imaging revealed a clear core–shell structure (Figure 2c), and Western blot confirmed the presence of platelet membrane marker CD41 on PLSCZ (Figure 2d). Elemental mapping (EDS) demonstrated uniform distribution of enzyme-related elements in SCZ (Figure 2e), and confocal microscopy showed high co-localization (Pearson’s coefficient: 0.95) between the lipid and membrane components (Figure 2f and Figure S3), confirming successful assembly.

Co-encapsulation within ZIF-8 significantly enhanced enzymatic activity and stability. SCZ exhibited higher clearance efficiency of O_2_^−·^ compared to individually encapsulated enzymes (Figure 2g). Moreover, ZIF-8 shielding protected the enzymes from proteolytic degradation (Figure 2h) and preserved activity in plasma conditions (Figure S4), underscoring the advantage of spatial confinement for cascade catalysis and delivery.

Despite the drug delivery potential of ZIF-8, its inherent hydrophobicity raises biocompatibility concerns, including reported cytotoxicity [48,49]. Existing chemical modification strategies often impair ZIF-8’s structural integrity, causing pore blockage that hinders drug diffusion. Thus, an ideal modification should selectively hydrophilize only the external surface while preserving internal porosity [50]. In this study, cytotoxicity assays using SH-SY5Y cells demonstrated that ZIF-8 coated with a hybrid platelet membrane exhibited significantly reduced cytotoxicity (Figure S5). Furthermore, the coating effectively suppressed hemolytic activity (Figure S6), confirming the crucial role of the hybrid platelet membrane in enhancing the biocompatibility of ZIF-8 for this nanoplatform application.

### 3.2. SCZ Exerts Neuroprotective Effects Through Anti-Oxidative Stress

**Figure 3:**
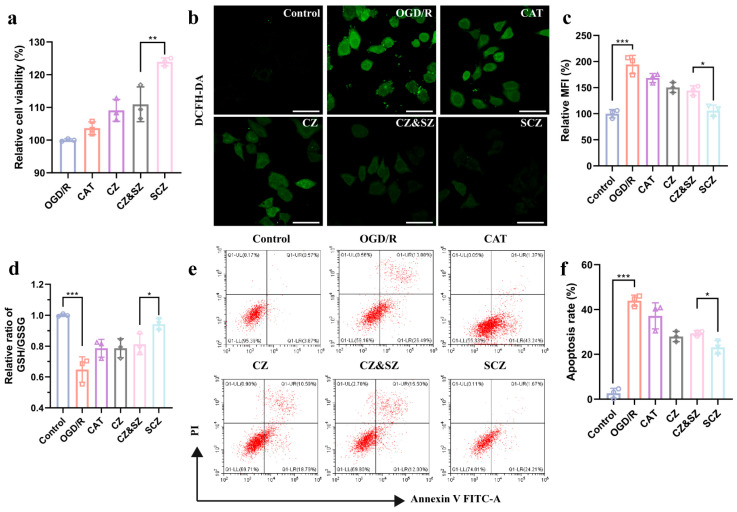
Neuroprotective effects against oxidative stress and apoptosis. (**a**) The cell viability of SH-SY5Y cells by treated with different nanoparticle formulations. (**b**) Representative CLSM image of intracellular reactive oxygen species content labeled by DCFH-DA. (**c**) Quantitative results of intracellular reactive oxygen species measured by flow cytometry using DCFH-DA. (**d**) The ratio of intracellular GSH to GSSG after different treatments. (**e**) Flow cytometry analysis images of cell apoptosis, gating on Annexin V-FITC/PI staining, and (**f**) quantified results. Data presented as mean ± SD (*n* = 3); * *p* < 0.05, ** *p* < 0.01, and *** *p* < 0.001.

Reperfusion injury is primarily driven by oxidative stress upon restored oxygen supply, leading to excessive ROS generation, which directly damages neurons and contributes to cell death [51]. It is precisely based on this pathological characteristic that an integrated antioxidant core SCZ was designed. To evaluate whether SCZ could counteract this oxidative damage and exert neuroprotection, we first utilized an OGD/R model in SH-SY5Y cells to evaluate the protective effect of SCZ. It was found that 53.68 ± 1.01% of the cells survived after 5 h of OGD followed by 24 h of reoxygenation (Figures S7 and S8). SCZ treatment significantly increased cell viability to 123.9 ± 1.0%, surpassing both that in the injured control (OGD/R) and physical mixture of separately encapsulated enzymes (SZ&CZ) (Figure 3a), demonstrating enhanced neuroprotection.

To further elucidate the underlying mechanism, we systematically assessed multiple markers of oxidative damage and cellular defense. By using the DCFH-DA probe [52], we demonstrated that the markedly elevated intracellular ROS by OGD/R was significantly attenuated by SCZ treatment (Figure 3b,c). Additionally, inducible nitric oxide synthase (iNOS) was further investigated, which is a key oxidative stress indicator whose pathological upregulation fuels a damaging cascade yielding peroxynitrite (ONOO−) from NO and O_2_^−·^ [53,54]. SCZ significantly downregulated iNOS (Figure S9), demonstrating the suppression of oxidative damage. While the intracellular concentration of ROS is typically controlled by cellular antioxidant defenses, these systems can become insufficient or overwhelmed under pathological conditions, resulting in the accumulation of free ROS and triggering oxidative injury [55]. To determine the effect of SCZ on cellular antioxidant capacity under such conditions, the glutathione/glutathione disulfide (GSH/GSSG) ratio, a central redox couple critical for oxidative stress resistance and major cellular antioxidant defenses [56,57,58], was subsequently measured. SCZ potently elevated this ratio (Figure 3d), demonstrating its role in restoring the endogenous antioxidant capacity. Ultimately, SCZ treatment significantly reduced late apoptosis of cells (Figure 3e,f), surpassing that in the SZ and CZ group, which is probably attributed to its superior antioxidant capacity in mitigating oxidative damage and restoring redox homeostasis, underscoring its crucial role in preserving neuronal viability under ischemic conditions.

### 3.3. Cellular Uptake and Intracellular Processes

**Figure 4:**
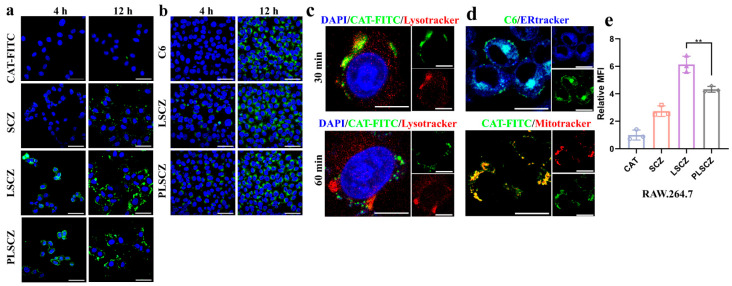
Cellular internalization and intracellular trafficking of PLSCZ. (**a**) Representative CLSM image of uptake of different formulations by SH-SY5Y cells (blue: DAPI for nucleus; green: FITC-CAT; and scale bar: 50 µm). (**b**) Representative CLSM image of uptake of different formulations by SH-SY5Y cells (blue: DAPI for nucleus; green: C6; and scale bar: 50 µm). (**c**) Representative CLSM images of colocalization analysis with lysosomes (30 vs. 60 min) (blue: DAPI for nucleus; green: CAT-FITC; red: Lysotracker; scale bar: 20 µm). (**d**) Representative CLSM images of targeting analysis of mitochondrial and ER (blue: ER tracker; green (up): CAT-FITC; green (down): C6; red: lysosome tracker; and scale bar: 40 µm). (**e**) The relative cell uptake rate of RAW 264.7 cells after different treatments after 12 h, determined by flow cytometry. Data presented as mean ± SD (*n* = 3); ** *p* < 0.01.

To elucidate the cellular internalization and intracellular trafficking of the nanoparticles, we examined their uptake in SH-SY5Y and BV2 cells using FITC-labeled CAT and the rapamycin surrogate C6. Uptake was time-dependent, with significantly higher accumulation at 12 h compared to 4 h (Figure 4a,b and Figures S10–S15). PLSCZ and LSCZ showed superior cellular uptake over SCZ and free C6, underscoring the role of the lipid membrane in facilitating entry. Furthermore, enzyme encapsulation within ZIF-8 (SCZ) markedly enhanced cellular uptake compared to free CAT.

Subcellular localization studies revealed rapid lysosomal uptake within 30 min (Pearson’s coefficient: 0.93 ± 0.01), followed by efficient lysosomal escape by 60 min (Pearson’s coefficient: 0.63 ± 0.07; *p* < 0.001) (Figure 4c and Figure S16), essential for preserving enzymatic activity. At 240 min, we innovatively discovered that SCZ accumulated predominantly near mitochondria (Pearson’s coefficient: 0.87 ± 0.03), while C6 localized in the endoplasmic reticulum (Pearson’s coefficient: 0.86 ± 0.03) (Figure 4d and Figures S17 and S18), indicating the potential ability of targeted disassembly of PLSCZ and mitochondrial proximity for ROS protection.

Uptake mechanisms were primarily mediated by clathrin and caveolin pathways. The fused membrane coating further promoted caveolin-dependent uptake and macropinocytosis in BV2 cells (Figures S19 and S20). Importantly, PLSCZ exhibited reduced uptake in RAW264.7 macrophages compared to LSCZ (Figure 4e), confirming CD47-mediated immune evasion and potential for prolonged circulation [59].

### 3.4. Mitochondrial Protection and Energy Recovery

**Figure 5:**
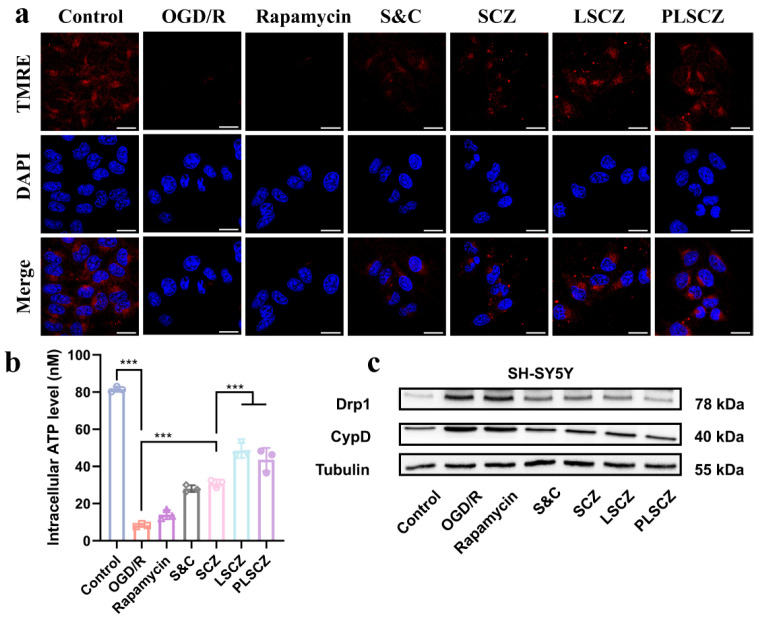
Mitochondrial protection and energy recovery. (**a**) Representative CLSM images of SH-SY5Y cells after different treatments (blue: DAPI for nucleus; red: TMRE for MMP determination; and scale bar: 20 µm). (**b**) Intracellular ATP content of SH-SY5Y cells. (**c**) Western blot of Cypd and Drp1 expression of SH-SY5Y cells. Data presented as mean ± SD (*n* = 3); *** *p* < 0.001.

Mitochondrial dysfunction, characterized by loss of membrane potential (ΔΨm) and impaired ATP synthesis, is a hallmark of cerebral ischemia–reperfusion injury [60,61]. We evaluated the ability of PLSCZ to restore mitochondrial function in SH-SY5Y and BV2 cells under OGD/R conditions. PLSCZ and LSCZ treatments significantly restored ΔΨm (Figure 5a and Figures S21–S23) and increased intracellular ATP levels (Figure 5b and Figure S24), with PLSCZ showing the greatest recovery due to enhanced cellular uptake and synergistic ROS scavenging.

At the molecular level, OGD/R induced elevated expression of Drp1 and CypD—key regulators of mitochondrial fission and permeability transition pore formation—indicating pathological mitochondrial remodeling [62,63]. PLSCZ and LSCZ effectively normalized their expression (Figure 5c and Figures S25–S29), correlating with functional recovery. These results demonstrate that PLSCZ preserves mitochondrial integrity by scavenging ROS, stabilizing mitochondrial membrane potential (ΔΨm), and inhibiting excessive fission, thereby promoting energy restoration and cellular survival.

### 3.5. Microglial Polarization and Anti-Inflammation In Vitro

**Figure 6:**
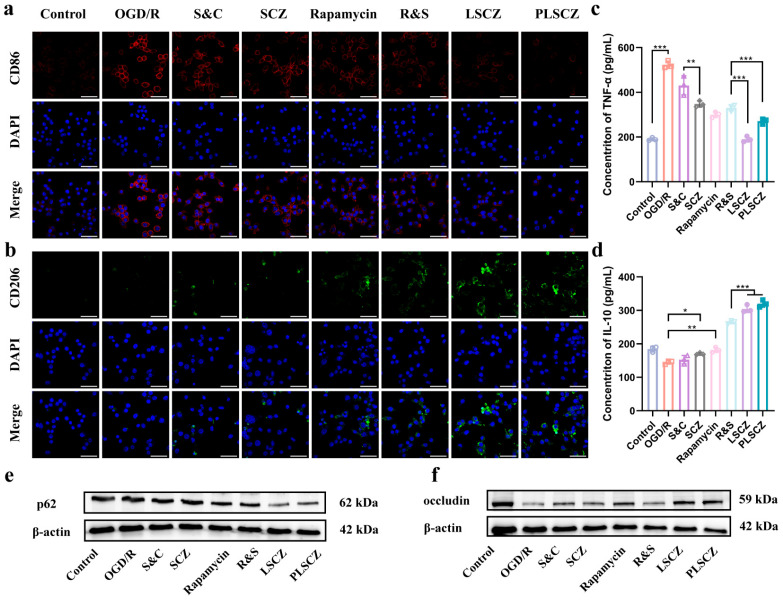
Microglial polarization and anti-inflammatory effects. (**a**) Representative CLSM images of BV2 cells after different treatments (blue: DAPI for nucleus; red: Anti-CD86-APC for M1 type label; and scale bar: 50 µm). (**b**) Representative CLSM images of BV2 cells after different treatments (blue: DAPI for nucleus; green: Anti-CD206-FITC for M2 type label; and scale bar: 50 µm). (**c**) The levels of TNF-α in BV2 cells. (**d**) The levels of IL-10 in BV2 cells. (**e**) Western blot of p62 expression in BV2 cells. (**f**) Western blot of occludin expression of bend.3 cells. Data presented as mean ± SD (*n* = 3); * *p* < 0.05, ** *p* < 0.01, and *** *p* < 0.001.

Neuroinflammation mediated by microglial polarization is a critical therapeutic target in ischemic stroke [64]. The pro-inflammatory polarization M1 type and the anti-inflammatory polarization M2 type have different effects on neuroinflammation [65,66,67]. Under OGD/R conditions, BV2 microglia exhibited a pro-inflammatory M1 phenotype, with elevated CD86 (M1 marker) and reduced CD206 (M2 marker) expression. Treatment with PLSCZ and LSCZ significantly reversed this trend, promoting a shift toward the reparative M2 phenotype (Figure 6a,b and Figures S30 and S31). This polarization was accompanied by decreased secretion of TNF-α and increased IL-10 (Figure 6c,d), indicating a robust anti-inflammatory response.

Moreover, the abnormality in autophagy flux in neurons after ischemic stroke caused a series of problems [68]. However, PLSCZ restored autophagic flux, as evidenced by enhanced degradation of p62 (Figure 6e and Figure S32), which is probably through rapamycin-mediated mTOR inhibition. This activation of autophagy contributed to reduced apoptosis and improved neuronal survival [69,70,71,72]. Furthermore, through the transwell experiment, microglial cells inhibited the downregulation of the tight junction protein occludin in b.End.3 cells (Figure 6f and Figure S33), promoting the repair of the blood–brain barrier. This might be due to the enhanced expression of IL-10 caused by the M2 polarization of microglial cells [73].

### 3.6. Brain Occlusions Targeting Ability and Endothelial Repair of PLSCZ

**Figure 7:**
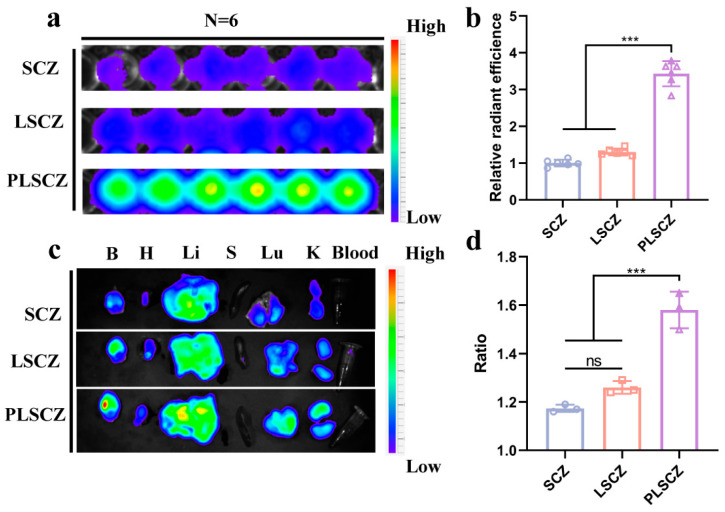
Targeting capability of PLSCZ to the ischemic hemisphere. (**a**) Representative image of nanoparticles binding with in vitro clots and (**b**) quantification results (*n* = 6). (**c**) IVIS imaging of major organs (*n* = 3). (**d**) Quantification of the fluorescence intensity ratio of both hemispheres (*n* = 3). Data presented as mean ± SD; ns *p* > 0.05, *** *p* < 0.001.

Leveraging the innate targeting properties of platelet membranes [28], PLSCZ exhibited strong binding to injured vasculature in vitro, demonstrating significantly higher adhesion to artificial thrombi compared to its non-coated counterparts (SCZ and LSCZ) (Figure 7a,b). This enhanced targeting is probably attributable to the presence of functional platelet membrane proteins, such as the collagen receptors α2β1 and GPVI, which mediate firm platelet adhesion [30,31,32], thereby conferring thrombus-targeting ability to PLSCZ.

The targeting capability of PLSCZ was further validated in a transient middle cerebral artery occlusion/reperfusion (tMCAO/r) rat model. PLSCZ exhibited significantly higher accumulation in the brain—1.7-fold and 1.6-fold greater than SCZ and LSCZ, respectively (Figure 7c and Figure S34). Importantly, comparison of the fluorescence intensity between the ischemic and healthy hemispheres revealed a significantly higher ipsilateral-to-contralateral ratio for PLSCZ (Figure 7d), validating its specific targeting capability to the injured vasculature. These in vivo findings align with the in vitro thrombus-binding assays, together demonstrating the specific affinity of PLSCZ for thrombus-associated endothelium. Furthermore, PLSCZ effectively crossed OGD/R-injured b.End.3 monolayers while remaining largely impermeable to intact barriers in a Transwell assay (Figure S35), which confirms its ability to leverage impaired BBB integrity for enhanced brain penetration and accumulation in the ischemic region. PLSCZ enables targeted ischemic brain delivery through platelet membrane-mediated vascular adhesion and impaired BBB penetration, underpinning its enhanced therapeutic efficacy against cerebral ischemia—reperfusion injury.

### 3.7. Evaluation of Neuroprotective Effect

**Figure 8:**
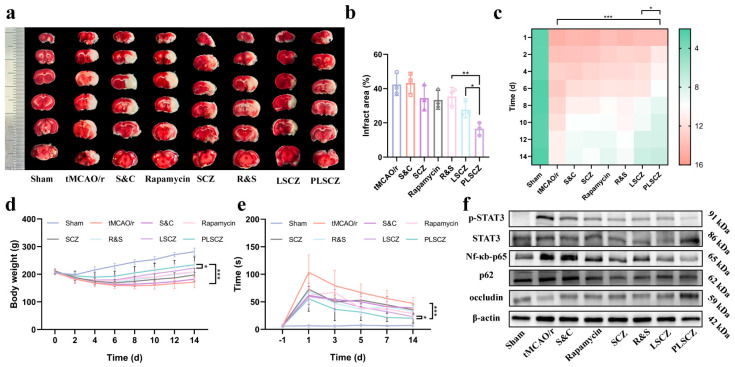
In vivo neuroprotection and functional recovery. (**a**) Representative TTC staining image of the brain slices and (**b**) quantification results. (*n* = 3) (**c**) The mNSS neurofunctional scores of tMCAO/r model rats after different treatments at different times (*n* = 6). (**d**) Weight change in rats (*n* = 6). (**e**) Sticky object removal time (*n* = 6). (**f**) Western blot of p-STAT3, STAT3, NF-κb-p65, p62, and occludin expression in brain tissues. Data presented as mean ± SD; * *p* < 0.05, ** *p* < 0.01, and *** *p* < 0.001.

For in vivo evaluation, a tMCAO/r rat model was successfully conducted (Figure S36). PLSCZ administration at reperfusion significantly attenuated cerebral ischemia–reperfusion injury. PLSCZ reduced infarct volume from 34.52 ± 6.03% (tMCAO/r) to 16.51 ±3.02% (Figure 8a,b), improved neurological function (mNSS score was improved from 10.83 ± 1.06 to 7.16 ± 1.46), and mitigated weight loss (Figure 8c–e). Mechanistic studies revealed that PLSCZ inhibited key inflammatory pathways, notably reducing the p-STAT3/STAT3 ratio and NF-κB p65 expression (Figure 8f and Figures S37 and S38), which correlated with the inflammatory process [74,75,76,77,78]. Furthermore, PLSCZ promoted the restoration of autophagic flux via p62 degradation (Figure 8f and Figure S39) and enhanced blood–brain barrier integrity through occludin upregulation (Figure 8f and Figure S40).

**Figure 9:**
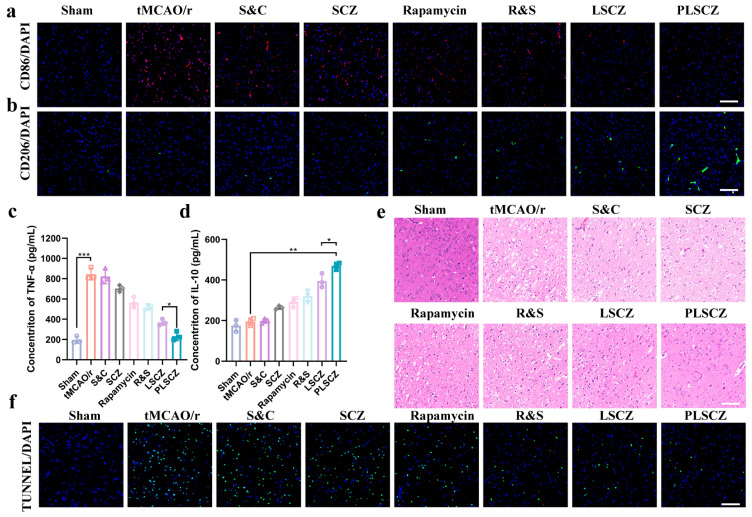
Modulation of neuroinflammation and neuronal survival. (**a**) Immunostaining of CD86 (M1 marker) (red: CD86; blue: DAPI; and scale bar: 100 µm). (**b**) Immunostaining of CD206 (M2 marker) (green: CD206; blue: DAPI; and scale bar: 100 µm). (**c**) The levels of TNF-α in the infarct area (*n* = 3). (**d**) The levels of IL-10 in the infarct area (*n* = 3). (**e**) H&E staining (Microglia marker) (scale bar: 100 µm). (**f**) Immunostaining of TUNEL (green: TUNEL; blue: DAPI; and scale bar: 100 µm). Data presented as mean ± SD; * *p* < 0.05, ** *p* < 0.01, and *** *p* < 0.001.

Immunohistochemical analysis indicated that PLSCZ modulated microglial polarization toward the protective M2 phenotype (reduced CD86+, increased CD206+, Figure 9a,b), leading to the decreased levels of pro-inflammatory cytokines (TNF-α) and elevated anti-inflammatory cytokines (IL-10) (Figure 9c,d). It also suppressed the activation of astrocytes (reduced GFAP) (Figure S41), which amplify neuroinflammatory responses if over-activated, worsening brain injury [79,80,81]. The reduction in microglial cell activation (reduced Iba-1) suggests a reduction in inflammation (Figure S42). Consequently, PLSCZ treatment reduced neuronal apoptosis and necrosis (Figure 9e,f), preserving neuronal viability (increased NeuN) (Figure S43).

## 4. Discussion

CIRI presents a formidable therapeutic challenge due to the intertwined pathologies of oxidative stress and neuroinflammation [4,5,6], which collectively drive secondary brain damage [7,8]. Although various antioxidant strategies have been developed to mitigate oxidative damage, they often prove inferior to natural enzymes, typically limited by insufficient ROS scavenging efficiency, short half-life, potential long-term toxicity, or rapid systemic clearance [13,14,15,16]. While natural antioxidant enzymes such as SOD and CAT offer a potent biological defense mechanism by operating in a concerted catalytic cascade to neutralize ROS [17,18]—a key driver of neuronal death, neuroinflammation, and blood–brain barrier disruption [10,11]—their clinical translation has been hampered by intrinsic instability, short half-life, and inefficient cascade kinetics [19]. Our findings demonstrate that PLSCZ not only enhances enzymatic stability and catalytic efficiency, thereby potently scavenging reactive oxygen species and mitigating oxidative damage, but also leverages its biomimetic coating to achieve precise targeting to the injured brain tissue, enabling a synergistic dual-pathway intervention against both oxidative and inflammatory injuries and thereby offering a comprehensive therapeutic strategy for CIRI.

The remarkable performance of PLSCZ is partly attributed to the superior properties of MOFs for enzyme immobilization, which demonstrate significant promise due to their tunable porosity, high specific surface area, and exceptional encapsulation capacity [20,21]. The co-encapsulation of SOD and CAT within ZIF-8 constitutes the functional core of the antioxidant activity of PLSCZ. Unlike free enzymes or their simple mixtures, the confined porous structure of ZIF-8 creates a nanoscale compartment that facilitates substrate channeling between the enzymes. This spatial co-confinement remarkably amplifies the overall cascade efficiency by minimizing the diffusion barrier for reaction intermediates [25,26], leading to significantly enhanced ROS degradation. This architectural and mechanistic advantage was directly evidenced by the superior O_2_^−·^ scavenging capacity of SCZ compared to individually encapsulated enzymes or their physical mixture (Figure 2g). Moreover, SCZ preserved enzyme activity in serum and effectively shielded the enzymes from proteolytic degradation (Figure 2h), leveraging protective mechanisms by the ZIF-8 shield, including the prevention of aggregation and proteolysis, and structural rigidification [82], thereby addressing a critical barrier for the in vivo application of protein-based therapeutics and informing rational strategies for the clinical delivery of macromolecular enzymes.

Subsequent cellular uptake experiments further demonstrated that PLSCZ exhibits significantly enhanced internalization capacity compared to free enzymes or free RAPA (Figure 4a,b), likely attributable to nanoparticle-mediated endocytosis [83]. Notably, the distinct intracellular distribution patterns of the C6-labeled lipid shell (representing rapamycin) and CAT-FITC (representing the SCZ core) post-lysosomal escape (Figure 4d) can be attributed to the progressive disintegration of PLSCZ. The ZIF-8 shell, known to be stable under neutral conditions but prone to degradation in acidic environments [84], likely facilitated lysosomal escape through a combination of pH-responsive dissolution and the proton-sponge effect [85], ultimately leading to the separation of the SCZ core from the lipid shell. Consequently, the liberated SCZ was enriched in mitochondria, enabling neutralization of ROS, which facilitates the highly efficient clearance of mitochondrially generated ROS. Critically, this mitochondrial enrichment of SCZ directly translated to functional recovery: PLSCZ treatment under OGD/R conditions significantly restored ΔΨm and intracellular ATP levels in SH-SY5Y and BV2 cells (Figure 5a,b and Figures S22–S24), which was further confirmed by normalized expression of key mitochondrial fission and permeability regulators Drp1 and CypD (Figure 5c and Figures S25–S29). Mitochondrial dysfunction is known to exacerbate brain injury in ischemic stroke patients; preserving mitochondrial function represents a vital aspect of IS treatment, a principle increasingly recognized in both contemporary neurology and traditional medicine approaches to neuroprotection [86].

A key innovation of PLSCZ lies in its functional surface engineering, where a hybrid platelet lipid membrane replaces conventional covalent modification to overcome biocompatibility and structural integrity limitations of MOF-based carriers [27,48,49,50]. Comprehensive characterization confirmed successful construction by tests of hydrodynamic diameter, zeta potential, core–shell morphology by TEM, preserved CD41 membrane protein, and co-localization (Figure 2b–f and Figures S2 and S3). This biomimetic design enabled active targeting to injured vasculature via GPIbα/CD47 (Figure 7a–d) while simultaneously reducing macrophage clearance, probably through CD47-mediated “don’t-eat-me” signaling (Figure 4e) [30,31,32]. Importantly, this surface modification strategy significantly enhanced the plasma stability of the ZIF-8-based carrier (Figure 2h and Figure S4), while leveraging the inherent properties of the platelet membrane to improve both targeting capability and immune evasion—attributes highly advantageous for effective drug delivery. Simultaneously, rapamycin loaded within the hybrid membrane promoted microglial M2 polarization, suppressed TNF-α, elevated IL-10, restored autophagic flux (Figure 6a–e), and ultimately improved neuronal survival, demonstrating synergistic antioxidant and anti-inflammatory efficacy against CIRI. Together, the integrated immunomodulation and antioxidant activity of PLSCZ provide a synergistic, dual-pathway intervention against CIRI.

The in vivo efficacy of PLSCZ was unequivocally demonstrated in a tMCAO/r rat model. Treatment with PLSCZ significantly reduced cerebral infarct volume, improved neurological scores, and facilitated somatosensory recovery and neuroprotection (Figure 8a–e, Figure 9e,f and Figure S43). Mechanistically, PLSCZ orchestrated a sequential amelioration of neuroinflammatory injury, a shift in microglial polarization from pro-inflammatory to anti-inflammatory phenotypes (Figure 9a,b), accompanied by suppressed astrocyte activation (Figure 9 and Figures S41 and S42). These cellular changes subsequently drove the downregulation of key inflammatory pathways, particularly STAT3 and NF-κB (Figure 8f), which in turn reduced pro-inflammatory cytokine production (Figure 9c,d). The attenuated neuroinflammatory milieu directly contributed to blood–brain barrier preservation, as definitively demonstrated by the upregulated expression of the tight junction protein occludin (Figure 8f). Based on these integrated findings, the in vivo studies substantiate that PLSCZ constitutes an effective and multifaceted intervention for alleviating cerebral ischemia–reperfusion injury.

Despite these promising results, several limitations warrant consideration. The translational potential of PLSCZ must be cautiously evaluated. First, while cell membrane-coated nanocarriers (including platelet membranes) show great promise [87,88], their scale-up faces practical challenges. Large-scale extraction of platelet membranes and batch-to-batch variability in nanoparticle preparation could impact reproducibility and clinical scalability. The most common method for platelet membrane extraction involves isolating it from whole blood through a series of mechanical processing steps [89]. Synthetic PM-mimetic strategies offer promising alternatives, though they must carefully preserve key targeting proteins [90]. Furthermore, recombinant technology provides a strategy by utilizing genetic engineering to efficiently express platelet-specific targeting proteins in prokaryotic or eukaryotic systems, which are then conjugated onto nanocarrier surfaces [91]. Platelet membranes contain various functional proteins essential for maintaining bioactivity [92]. In this context, methods for preserving platelet components—such as refrigeration, cryopreservation, or lyophilization—offer potential advantages by providing a prolonged shelf life and potentially improving safety profiles with a lower risk of bacterial contamination [93,94,95,96]. These strategies may help standardize the starting material and mitigate batch-to-batch variability. Second, the long-term biocompatibility and clearance pathways of ZIF-8 degradation products in the brain require further investigation. While our hybrid membrane coating mitigated acute cytotoxicity and hemolysis, a comprehensive toxicological profile is necessary.

## 5. Conclusions

In conclusion, this study introduces PLSCZ as a robust and multi-functional nanoplatform for the treatment of CIRI. By synergistically combining cascade antioxidant enzymes with anti-inflammation, PLSCZ effectively addresses the dual pathology of oxidative stress and neuroinflammation. Our findings validate the strategic design of biomimetic, MOF-based carriers for complex cerebrovascular diseases and highlight the importance of coordinated multi-pathway intervention. Future work will focus on optimizing the manufacturing process, conducting long-term biosafety evaluations, and further deciphering the intricate signaling networks involved in its therapeutic efficacy. This platform opens new avenues for developing advanced nanotherapeutics against ischemic stroke and other neuroinflammatory disorders.

## Data Availability

The data presented in this study are available on request from the corresponding authors upon request.

## Supplementary Materials

The following supporting information can be downloaded at: https://www.mdpi.com/article/10.3390/pharmaceutics17111478/s1.
