# Supplementary material for: MOF-Engineered Platelet-Mimicking Nanocarrier-Encapsulated Cascade Enzymes for ROS Scavenging and Anti-Inflammation in Cerebral Ischemia–Reperfusion Injury
Source: Pharmaceutics. 2025 Nov 16;17(11):1478. doi: 10.3390/pharmaceutics17111478 (PMC12655722; doi:10.3390/pharmaceutics17111478)
Supplement: Supplementary file 1 [file pharmaceutics-17-01478-s001.zip › pharmaceutics-3967091-supplementary.pdf]

Supplementary information

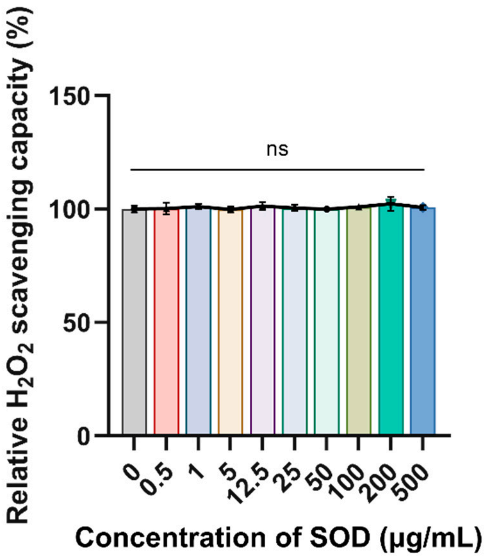

Figure S1. Relative H_2O_2 scavenging capacity of physical mixture of CAT/SOD with different mass ratio. (n=3, *P < 0.05, **P < 0.01, ***P < 0.001.)

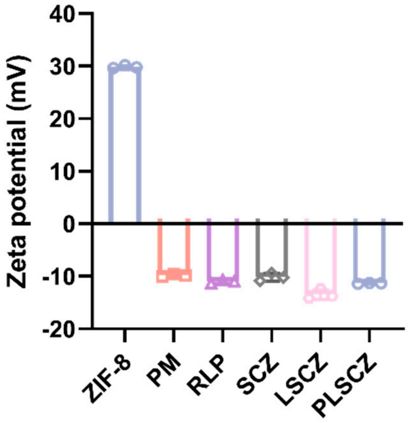

Figure S2. Zeta potential of different nanoparticles formulations. (n=3)

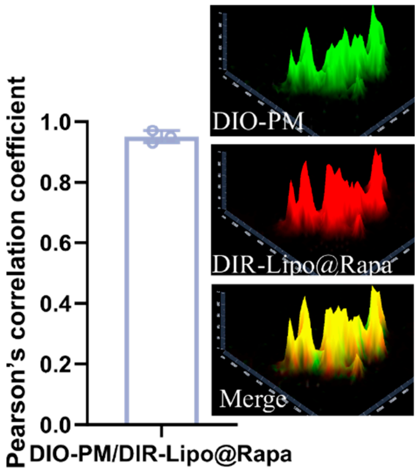

Figure S3. Co-localization analysis 2.5D plots and quantification. (n=3)

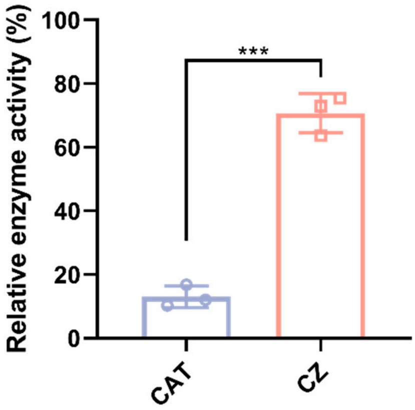

Figure S4. Serum stability assays. (n=3, *P < 0.05, **P < 0.01, ***P < 0.001.)

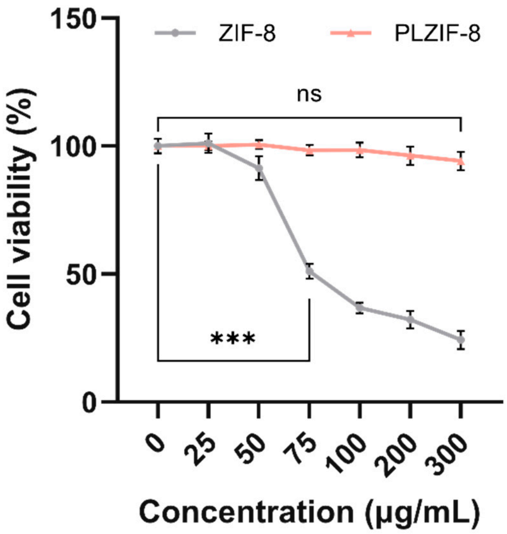

Figure S5. Cytotoxicity assays of ZIF-8 and PLZIF-8 using SH-SY5Y cells. (n=5, *P

< 0.05, **P < 0.01, ***P < 0.001.)

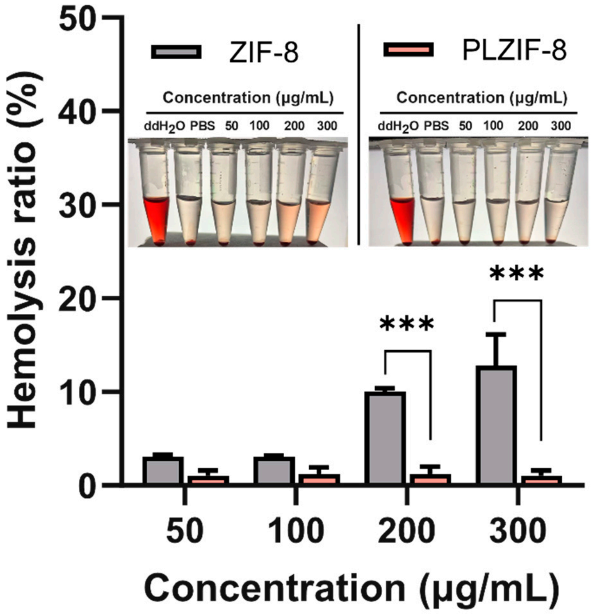

Figure S6. Hemolysis analysis of ZIF-8 and PLZIF-8. (n=5, *P < 0.05, **P < 0.01, ***P < 0.001.)

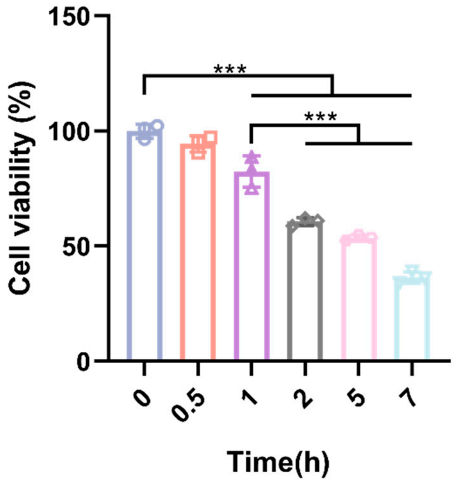

Figure S7. Cell viability under varying OGD durations. (n=3, *P < 0.05, **P < 0.01, ***P < 0.001.)

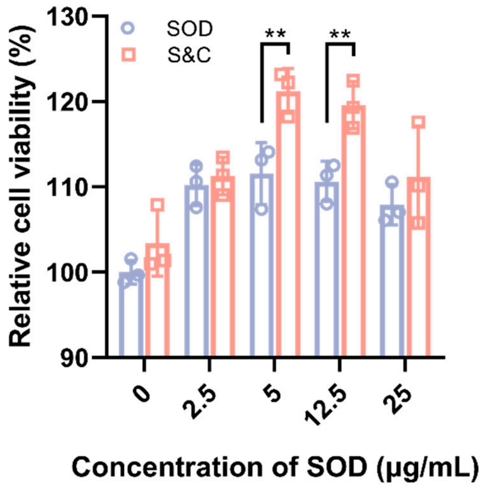

Figure S8. Dose-dependent neuroprotection by SOD/CAT combinations. (n=3, *P < 0.05, **P < 0.01, ***P < 0.001.)

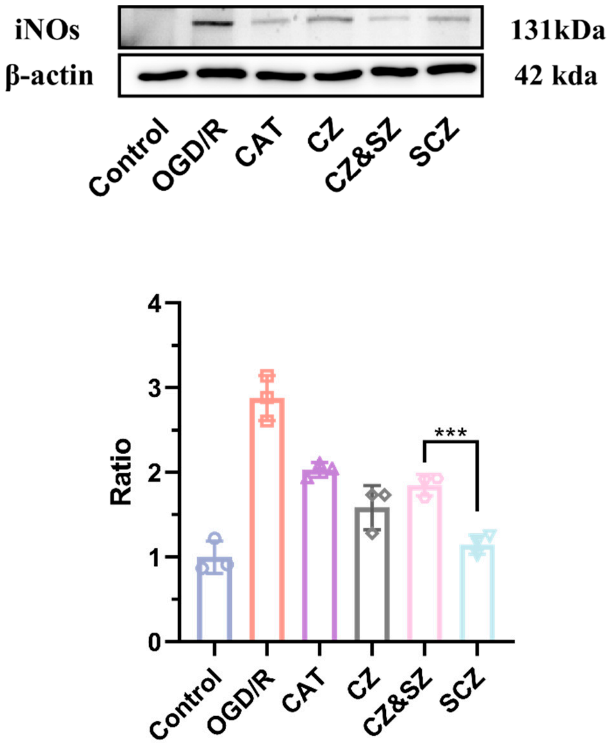

Figure S9. Western blot of iNOS expression and quantified results. (n=3, *P < 0.05, **P < 0.01, ***P < 0.001.)

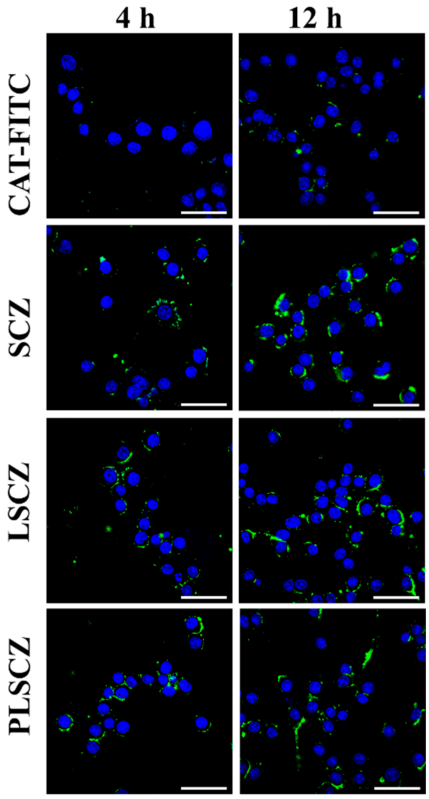

Figure S10. Representative CLSM image of uptake of different formulations by BV.2 cells. (blue: DAPI for nucleus; green: FITC-CAT; scale bar: 50 μm)

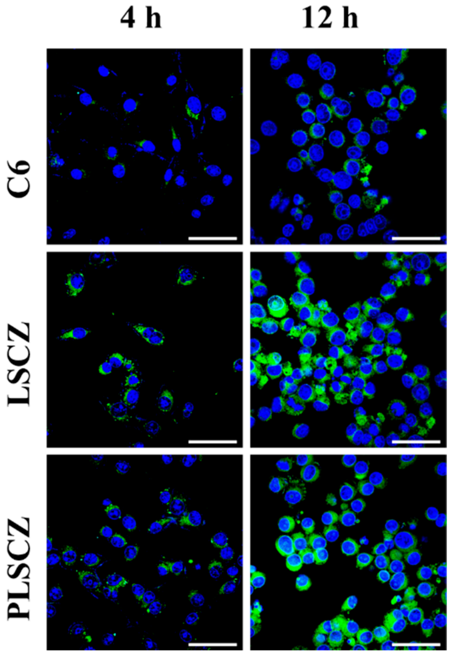

Figure S11. Representative CLSM image of uptake of different formulations by BV.2 cells. (blue: DAPI for nucleus; green: C6; scale bar: 50μm)

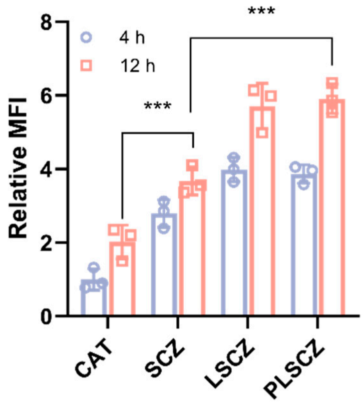

Figure S12. Quantified results of the uptake of different formulations by SH-SY5Y cells. (n=3, *P < 0.05, **P < 0.01, ***P < 0.001.)

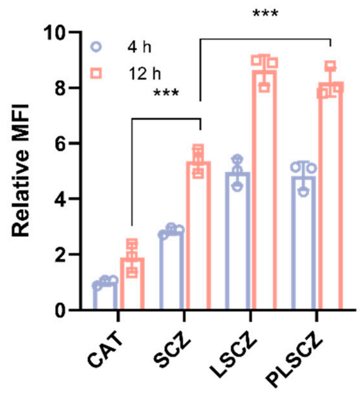

Figure S13. Quantified results of the uptake of different formulations by BV.2 cells. (n=3, *P < 0.05, **P < 0.01, ***P < 0.001.)

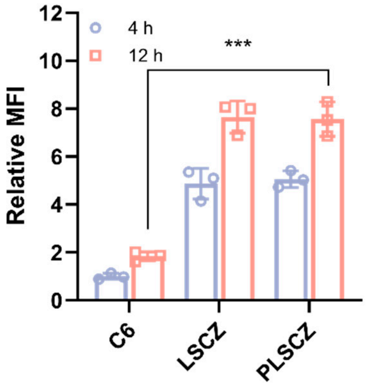

Figure S14. Quantified results of the uptake of different formulations by SH-SY5Y cells. (n=3, *P < 0.05, **P < 0.01, ***P < 0.001.)

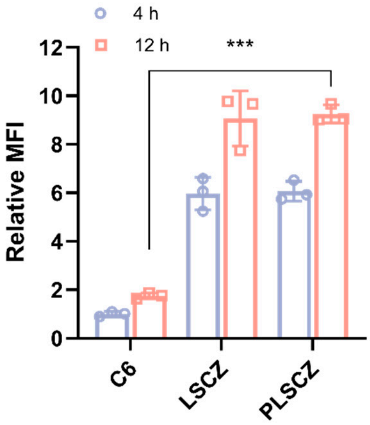

Figure S15. Quantified results of the uptake of different formulations by BV.2 cells. (n=3, *P < 0.05, **P < 0.01, ***P < 0.001.)

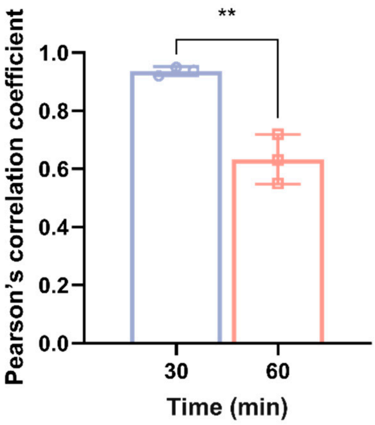

Figure S16. Pearson's correlation coefficient of CAT-FITC with lysosomes by CLSM (30 vs. 60 min). (n=3, *P < 0.05, **P < 0.01, ***P < 0.001.)

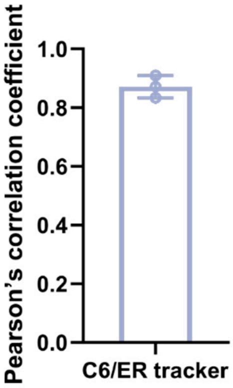

Figure S17. Pearson's correlation coefficient of C6 with ER by CLSM. (n=3)

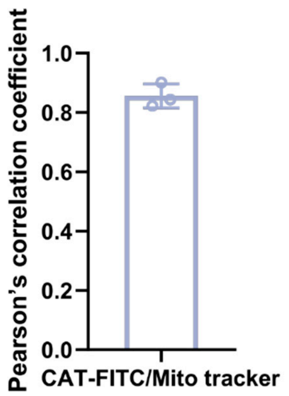

Figure S18. Pearson's correlation coefficient of CAT-FITC with mitochondrial by CLSM. (n=3)

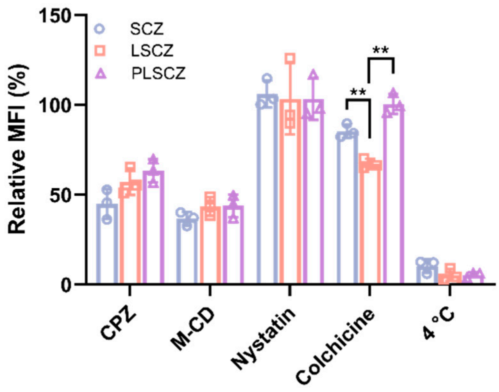

Figure S19. The relative cell uptake of BV.2 cells after different treatments determined by flow cytometry. (n=3, *P < 0.05, **P < 0.01, ***P < 0.001.)

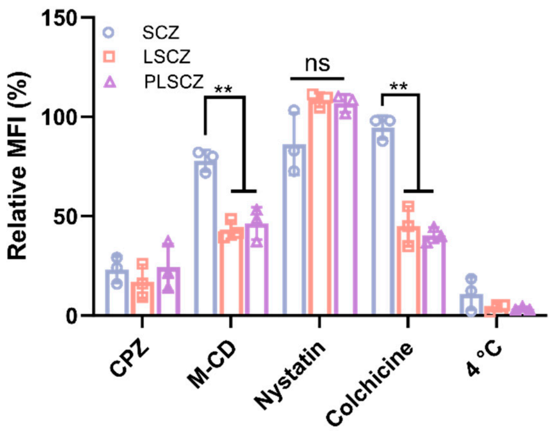

Figure S20. The relative cell uptake of SH-SY5Y cells after different treatments determined by flow cytometry. (n=3, *P < 0.05, **P < 0.01, ***P < 0.001.)

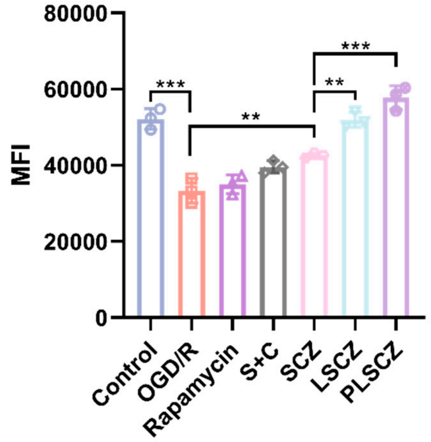

Figure S21. Flow cytometry results of TMRE determination of SH-SY5Y cells after different treatments (n=3, *P < 0.05, **P < 0.01, ***P < 0.001.)

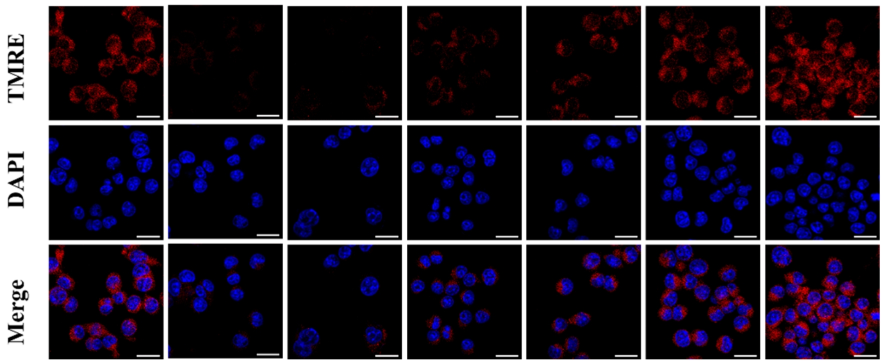

Figure S22. Representative CLSM images of BV.2 cells after different treatments (blue: DAPI for nucleus; red: TMRE for MMP determination; scale bar: 20 μm) (Group left to right:Control; OGD/R; Rapamycin, S&C; SCZ; LSCZ; PLSCZ)

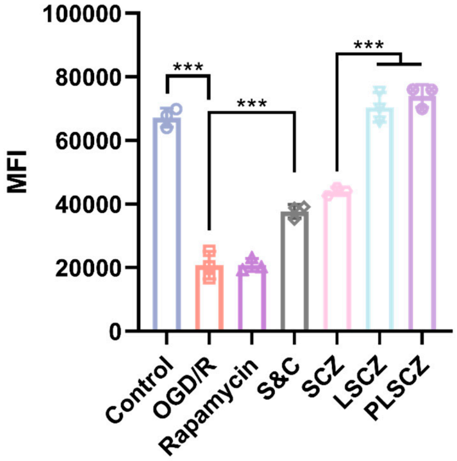

Figure S23. Flow cytometry results of TMRE determination of BV.2 cells after different treatments (n=3, *P < 0.05, **P < 0.01, ***P < 0.001.)

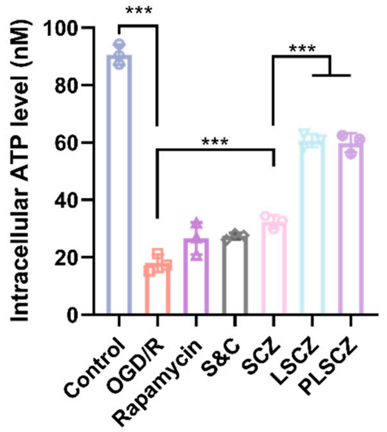

Figure S24. Intracellular ATP content of BV.2 cells. (n=3, *P < 0.05, **P < 0.01, ***P < 0.001.)

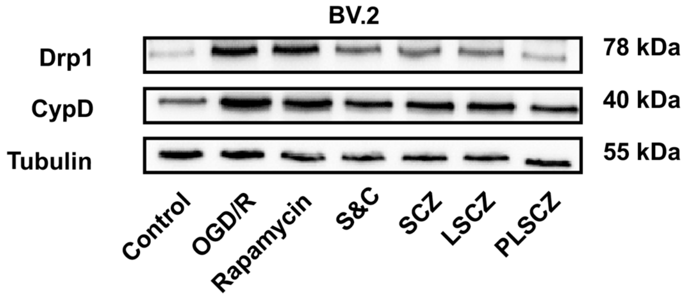

Figure S25. Western blot of Cypd and Drp1 expression of BV.2 cells (n=3, *P < 0.05, **P < 0.01, ***P < 0.001.)

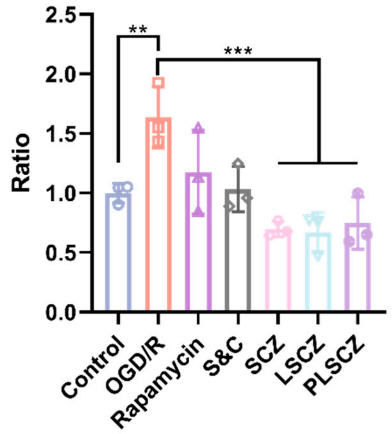

Figure S26. Quantified results of western blot of Cypd expression of SH-SY5Y cells (n=3, *P < 0.05, **P < 0.01, ***P < 0.001.)

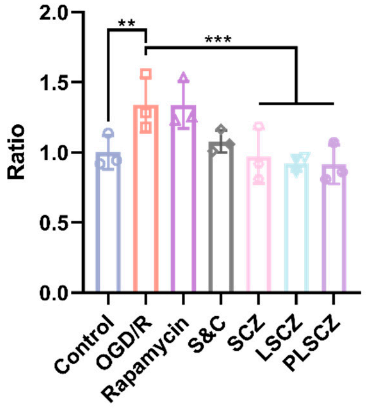

Figure S27. Quantified results of western blot of Drp1 expression of SH-SY5Y cells (n=3, *P < 0.05, **P < 0.01, ***P < 0.001.)

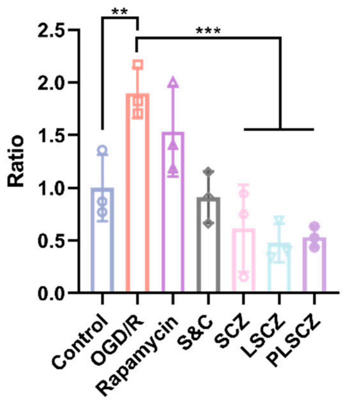

Figure S28. Quantified results of western blot of CYPD expression of BV.2 cells (n=3, *P < 0.05, **P < 0.01, ***P < 0.001.)

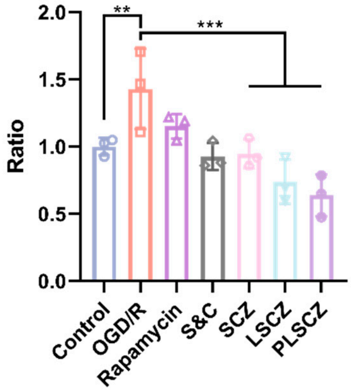

Figure S29. Quantified results of western blot of Drp1 expression of BV.2 cells (n=3, *P < 0.05, **P < 0.01, ***P < 0.001.)

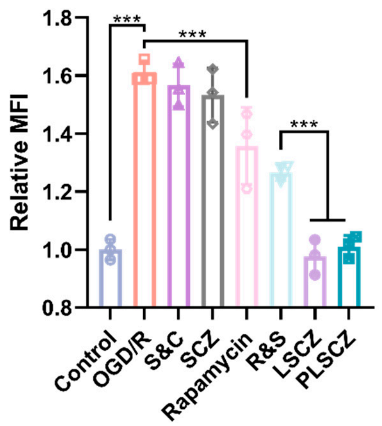

Figure S30. Flow cytometry results of CD86 immunostaining of BV.2 cells after different treatments. (n=3, *P < 0.05, **P < 0.01, ***P < 0.001.)

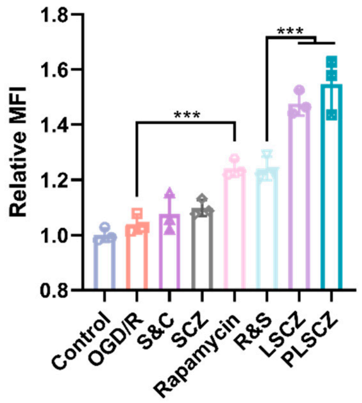

Figure S31. Flow cytometry results of CD206 immunostaining of BV.2 cells after different treatments. (n=3, *P < 0.05, **P < 0.01, ***P < 0.001.)

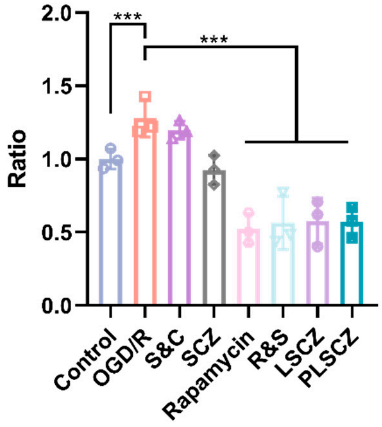

Figure S32. Quantified results of p62 expression of BV.2 cells. (n=3, *P < 0.05, **P < 0.01, ***P < 0.001.)

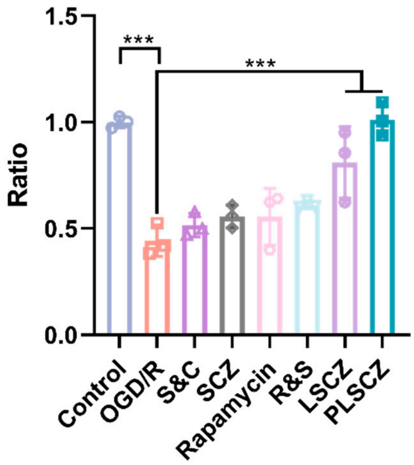

Figure S33. Quantified results of occludin expression of bend.3 cells. (n=3, *P < 0.05, **P < 0.01, ***P < 0.001.)

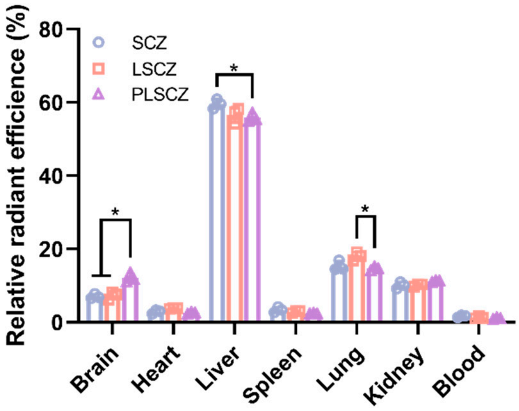

Figure S34. Quantification results of IVIS imaging of major organs. (n=3, *P < 0.05, **P < 0.01, ***P < 0.001.)

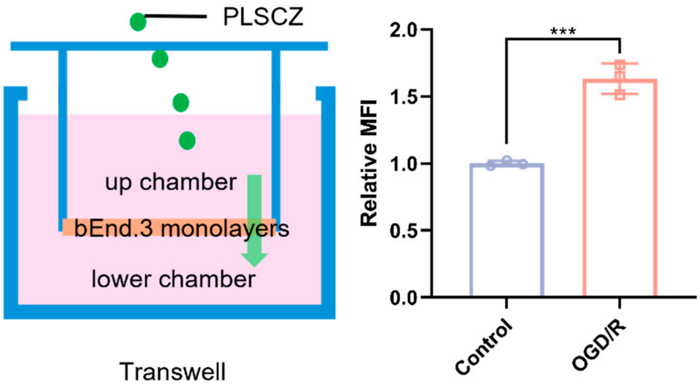

Figure S35. BBB permeability assay using transwell model. (n=3, *P < 0.05, **P < 0.01, ***P < 0.001.)

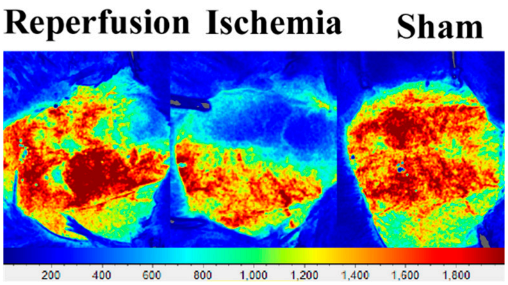

Figure S36. Representative image of laser speckle flow imaging of rat before and after MCAO surgery. (n=3, *P < 0.05, **P < 0.01, ***P < 0.001.)

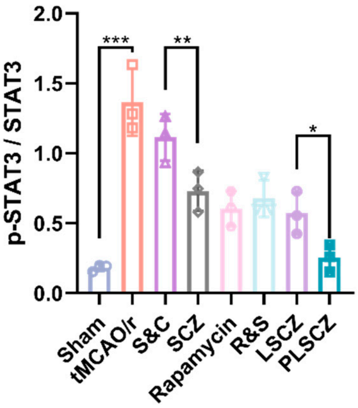

Figure S37. Quantified results of Western blot of p-STAT3/STAT3. (n=3, *P < 0.05, **P < 0.01, ***P < 0.001.)

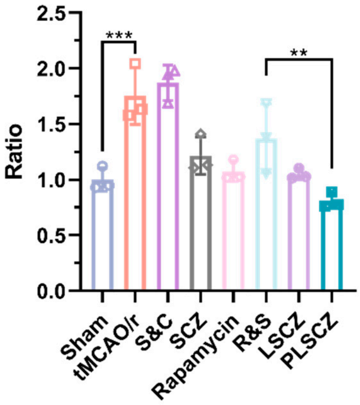

Figure S38. Quantified results of Western blot of NF-b-p65. (n=3, *P < 0.05, **P < 0.01, ***P < 0.001.)

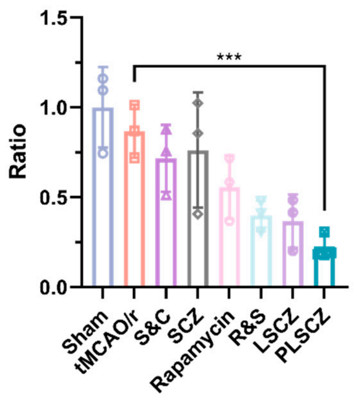

Figure S39. Quantified results of Western blot of p62. (n=3, *P < 0.05, **P < 0.01, ***P < 0.001.)

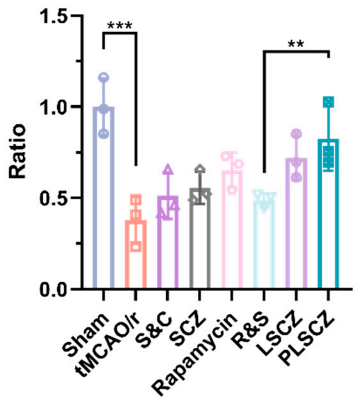

Figure S40. Quantified results of Western blot of occludin. (n=3, *P < 0.05, **P < 0.01, ***P < 0.001.)

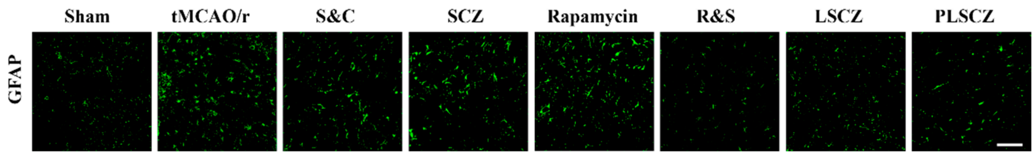

Figure S41. Immunostaining of GFAP (Astrocytes marker) (green: GFAP; scale bar: 100 µm)

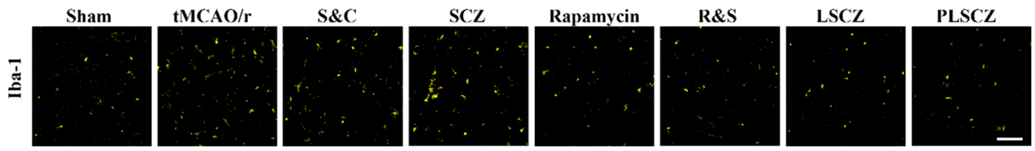

Figure S42. Immunostaining of Iba-1 (Microglia marker) (yellow: Iba-1; scale bar: 100 µm)

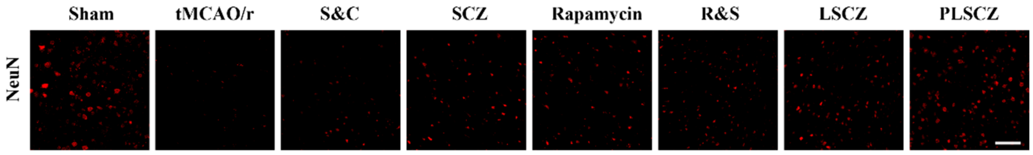

Figure S43. Immunostaining of NeuN (Neuron marker) (red: NeuN; scale bar: 100 μm).

Original western blot images

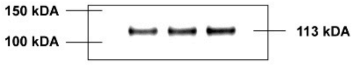

Figure S44. Original western blot image of fig 2d CD41

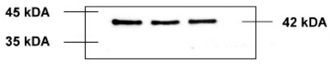

Figure S45. Original western blot image

of fig 2d β-actin

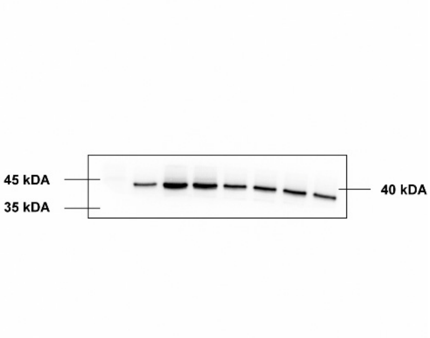

Figure S46. Original western blot image of fig 5c CYPD

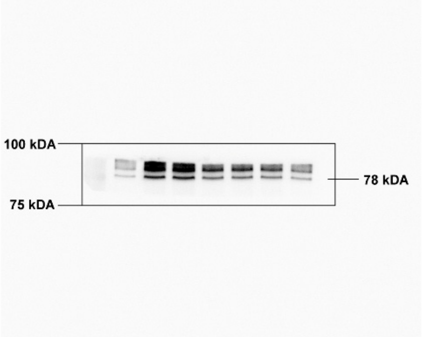

Figure S47. Original western blot image of fig 5c drp1

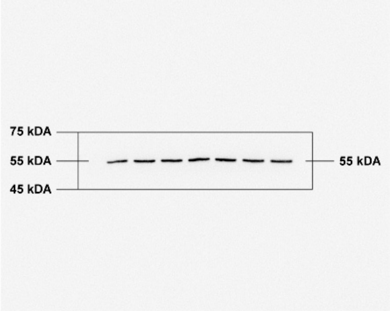

Figure S48. Original western blot image of fig 5C Tublin

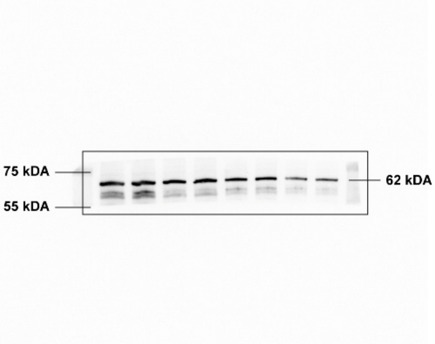

Figure S49. Original western blot image of fig 6e p62

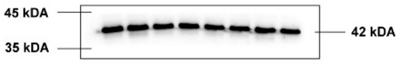

Figure S50. Original western blot image

of fig 6e β-actin

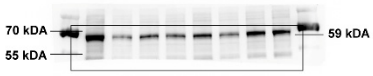

Figure S51. Original western blot image of fig 6f occludin

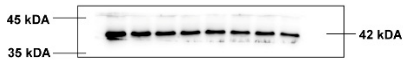

Figure S52. Original western blot image of fig 6f β-actin

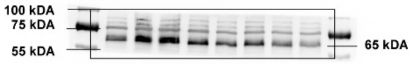

Figure S53. Original western blot image of fig 8f Nfκb p65

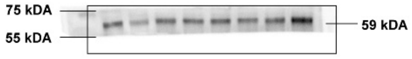

Figure S54. Original western blot image of fig 8f occluding

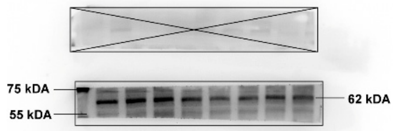

Figure S55. Original western blot image of fig 8f p62

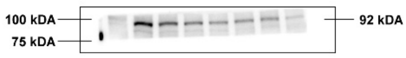

Figure S56. Original western blot image of fig 8f p-stat3

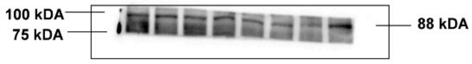

Figure S57. Original western blot image of fig 8f Stat3

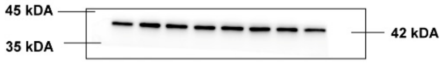

Figure S58. Original western blot image of fig 8f β-actin

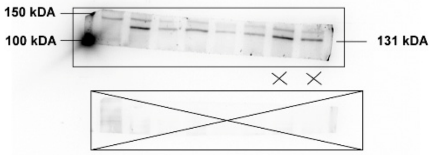

Figure S59. Original western blot image of fig S8 iNOs

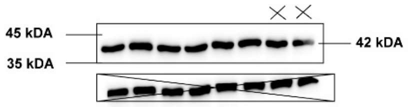

Figure S60. Original western blot image of fig S8 β-actin

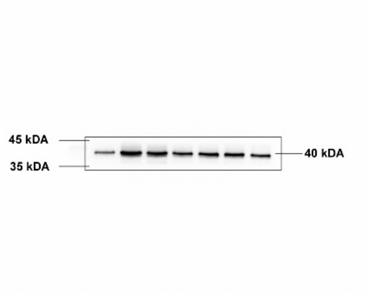

Figure S61. Original western blot image of fig S25 CYPD

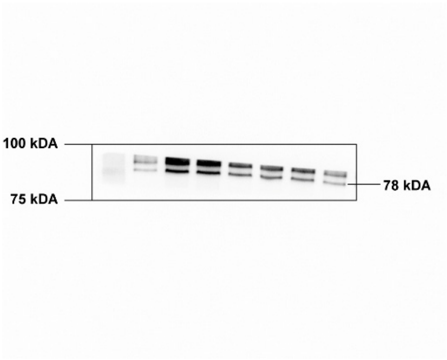

Figure S62. Original western blot image of fig S25 drp1

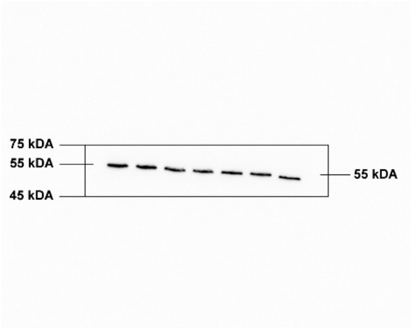

Figure S63. Original western blot image of fig S25 Tublin
